# Exploring sequence- and structure-based fitness landscapes to enhance thermal resistance and activity of endoglucanase II with minimal experimental effort†

**DOI:** 10.1039/d5cb00013k

**Published:** 2025-05-05

**Authors:** Atul Kumar, Alexander-Maurice Illig, Nicolas de la Vega Guerra, Francisca Contreras, Mehdi D. Davari, Ulrich Schwaneberg

**Affiliations:** a Lehrstuhl für Biotechnologie, RWTH Aachen University, Worringerweg 3 52074 Aachen Germany u.schwaneberg@biotec.rwth-aachen.de; b Department of Bioorganic Chemistry, Leibniz Institute of Plant Biochemistry, Weinberg 3 06120 Halle Germany

## Abstract

Enhancing the performance of cellulases at high temperatures is crucial for efficient biomass hydrolysis—a fundamental process in biorefineries. Traditional protein engineering methods, while effective, are time-consuming and labour-intensive, limiting rapid advancements. To streamline the engineering process, we tested two distinct *in silico* methods for predicting thermally resistant and highly active variants of *Penicillium verruculosum* endoglucanase II. Specifically, we used FoldX to pinpoint structure-stabilizing substitutions (ΔΔ*G* < 0) and applied the sequence-based method EVmutation to identify evolutionarily favorable substitutions (Δ*E* > 0). Experimental validation of the top 20 ranked single-substituted variants from both methods showed that EVmutation outperformed FoldX, identifying variants with enhanced enzyme activity after one-hour incubation at 75 °C (up to 3.6-fold increase), increased melting temperature (Δ*T*_m_ of 2.8 °C), and longer half-lives at 75 °C (up to 104 minutes *vs.* 40 minutes for the wild type). Building upon these results, EVmutation was used to predict variants with two amino acid substitutions. These double-substituted endoglucanase variants showed further improvements—up to a 4.4-fold increase in activity, Δ*T*_m_ gains of 3.7 °C, and half-life extensions up to 82 minutes. This study highlights EVmutation's potential for accelerating protein engineering campaigns and enhancing enzyme properties while reducing experimental efforts, thereby contributing to more efficient and sustainable bioprocesses.

## Abstract 2

EVmutation outperformed FoldX in predicting improved endoglucanase variants, accelerating enzyme engineering while minimizing experimental workload.

## Graphical abstract

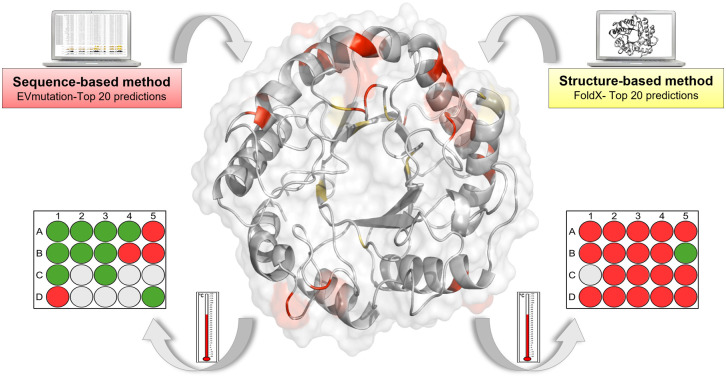

## Introduction

Cellulases are among the most important enzyme classes in the biorefinery industry, playing a crucial role in the sustainable production of chemicals, commodities, and biofuels. The global market for biorefinery products is projected to grow from $775.2 billion in 2024 to $1.2 trillion by 2029, reflecting a compound annual growth rate of 8.8%.^1^ Cellulases are pivotal for breaking down cellulose, which accounts for approximately 40–50% of the dry weight of lignocellulosic biomass.^2,3^ Three types of cellulases—endoglucanases (EC 3.2.1.4), cellobiohydrolases (EC 3.2.1.91) and β-glucosidases (EC 3.2.1.21)—work synergistically to depolymerize cellulose into sugar monomers.^4–8^ These monomers can then be further derivatized or used to produce biofuels through biochemical and chemocatalytic processes.^9–12^ Elevated temperatures accelerate the hydrolytic reaction by reducing the biomass recalcitrance.^13,14^ As a result, thermostable cellulases are the preferred choice for cost-effective hydrolysis of cellulosic biomass, supporting bioethanol production as part of the industrial bioeconomy.^13,15–19^

The performance of an enzyme at high temperatures is governed by its thermodynamic, kinetic, and process stability.^20–22^ Thermodynamic stability reflects the inherent strength of the enzyme's folded state and is measured by parameters such as the free energy of unfolding (Δ*G*), the unfolding equilibrium constant, and the melting temperature (*T*_m_).^23^ Kinetic stability indicates the enzyme's resistance to irreversible inactivation, and is measured by metrics such as the half-life of denaturation (*t*_1/2_), the deactivation constant, the optimum temperature, and the temperature at which the enzymatic activity is reduced by half (*T*_50_)^23^ Process stability, on the other hand, refers to the enzyme's ability to remain active throughout the reaction, as measured by the total turnover number and the productivity number.^22^

The function of enzymes hinges on a delicate balance between stability and activity. Optimizing enzymes for demanding industrial conditions, such as high temperatures, is challenging due to the intrinsic tradeoff between activity and stability.^24–26^ While thermal resistance demands a stable, rigid structure, sufficient flexibility is necessary to achieve enzymatic activity.^27^ Protein engineering enables the exploration of the protein's sequence space and enhancement of enzyme properties to meet specific application demands.^28,29^ It has been widely applied to improve the thermostability of cellulases, with various approaches, including directed evolution, rational design, and their combinations (see Table S1 in ESI†). A major challenge in engineering proteins for industrial processes is meeting time constraints. Traditional protein engineering campaigns, while effective, often involve multiple iterative rounds of directed evolution and numerous site-directed mutagenesis experiments making the process costly and time-consuming.^30^ Computational methods have the potential to accelerate protein engineering by predicting improved variants; however, they often require substantial data or detailed system knowledge to be effective. As a result, there is an increasing demand for efficient, user-friendly tools that facilitate protein engineering without requiring extensive computational expertise or large datasets.

In this study, we evaluated two computational protein engineering methods for predicting thermal resistant and catalytically active variants of endoglucanase II from *Penicillium verruculosum* (EGLII).^31–34^ Specifically, we employed a structure-based method (FoldX^35^) and a sequence-based method (EVmutation^36^). EGLII is classified as a member of subfamily 5 within the Glycoside Hydrolase family 5 (GH5) of cellulases. It has a characteristic (α/β)_8_ TIM barrel structure with eight β-sheets in the core surrounded by eight α-helices on the surface.^34,37,38^ Given EGLII's inherent thermal resistance, it presents a challenging test case for the comparison of methods to further enhance its thermal resistance.^31,32,39,40^

Using FoldX, we aimed to identify structure-stabilizing substitutions—variants with a single amino acid substitution that exhibit a greater free energy of unfolding compared to the wild type.^35^ This method has been widely employed to enhance protein thermal resistance, either independently or in combination with other techniques such as *B*-factor analysis or molecular dynamics (MD) simulations.^41–46^ In addition, we explored an alternative method that leverages information from a different data source. As genomic sequence databases expand at a faster rate than structural or biochemical databases, *in silico* methods that utilize amino acid sequences are becoming increasingly appealing. Models derived from direct coupling analysis (DCA) on multiple sequence alignments have demonstrated the ability to quantify the effects of predicted substitutions (Δ*E*). A study by Hopf *et al.* showed that Δ*E* correlates with protein fitness across a range of proteins and properties, leading to the development of a method known as EVmutation.^36^ Furthermore, a study demonstrated the effectiveness of DCA-based models in guiding the engineering of ketoisovalerate decarboxylase, increasing its *T*_50_ value up to 3.9 °C.^47^ Motivated by these findings, we decided to evaluate the performance of EVmutation in identifying substitutions that could enhance thermal resistance and activity of a cellulase.

To evaluate the effectiveness of both methods in identifying beneficial variants, we generated and characterized the top 20 predicted variants with a single substitution from each method in the laboratory. We assessed their enzymatic activity following thermal stress and determined their melting points and half-lives. Given the comparatively better performance of EVmutation in identifying improved variants with a single substitution and its high throughput, we expanded our investigation to include variants with two substitutions. We subsequently characterized the 20 variants exhibiting the highest Δ*E* in the laboratory. This study showcases the applicability, ease, and efficiency of a sequence-based *in silico* method for engineering thermal resistance and activity in cellulases, as demonstrated in our case study. By reducing experimental efforts and accelerating the development of robust enzymes, this method contributes to more sustainable bioprocesses.

## Results and discussion

In this study, we conducted a comparative investigation to enhance the thermal resistance and enzyme activity of EGLII using two distinct *in silico* methods: a structure-based method, FoldX,^35^ and a sequence-based method, EVmutation.^36^ We evaluated the performance of these methods by experimentally characterizing the top 20 predicted variants from each method. The assessment included enzymatic activity after thermal stress, melting points, and half-lives. Additionally, based on the results of single substitutions, we used EVmutation to predict beneficial variants with two substitutions. Unless otherwise specified, all values are expressed as mean ± standard error of the mean (SEM), based on at least three independent biological replicates.

### Prediction of beneficial substitutions

FoldX and EVmutation were employed to predict potentially beneficial substitutions for EGLII, aiming to enhance thermal resistance and increased enzyme activity. FoldX utilizes structural data along with a force field to assess differences in free energy of unfolding between the wild type and its variants. In contrast, EVmutation relies on a statistical model that assigns evolutionary probabilities to variants. Consequently, variants predicted to either exhibit increased free energy of unfolding (negative ΔΔ*G*), or high statistical energies (positive Δ*E*) compared to the wild type were selected for experimental validation (Table 1).

**Table 1:** Top 20 variants with a single substitution predicted by FoldX and EVmutation. The variants were ranked based on their differences in free energy of folding (ΔΔ*G*) and their differences in statistical energy (Δ*E*) between variant and wild type. Values of ΔΔ*G* are expressed as the mean ± SEM, based on five independent runs

Rank	FoldX		EVmutation	
	Variant	ΔΔ*G* (kcal mol^−1^)	Variant	Δ*E*
1	D138N	−5.35 ± 0.02	Q289G	3.13
2	D138C	−4.34 ± 0.01	V226I	2.63
3	E205M	−4.32 ± 0.08	P111I	2.42
4	D138L	−4.14 ± 0.01	D164A	2.34
5	E20M	−4.04 ± 0.27	A178S	1.97
6	E205C	−4.01 ± 0.01	P111V	1.72
7	D138V	−3.94 ± 0.37	Q289A	1.65
8	E205Q	−3.93 ± 0.06	S180T	1.26
9	E205L	−3.87 ± 0.01	T110N	1.22
10	S280L	−3.78 ± 0.30	N299S	1.18
11	S280M	−3.77 ± 0.01	E81A	1.00
12	D138A	−3.62 ± 0.01	D164G	0.99
13	D138S	−3.60 ± 0.03	T236Q	0.93
14	E20L	−3.36 ± 0.22	V150L	0.87
15	G181R	−3.32 ± 0.24	Y188V	0.70
16	S114D	−3.32 ± 0.01	T236A	0.68
17	D138T	−3.20 ± 0.09	Q239K	0.68
18	E175M	−3.09 ± 0.29	L201K	0.67
19	E205A	−3.05 ± 0.01	S123T	0.67
20	E20I	−2.84 ± 0.25	A153D	0.66

**Fig. 1:**
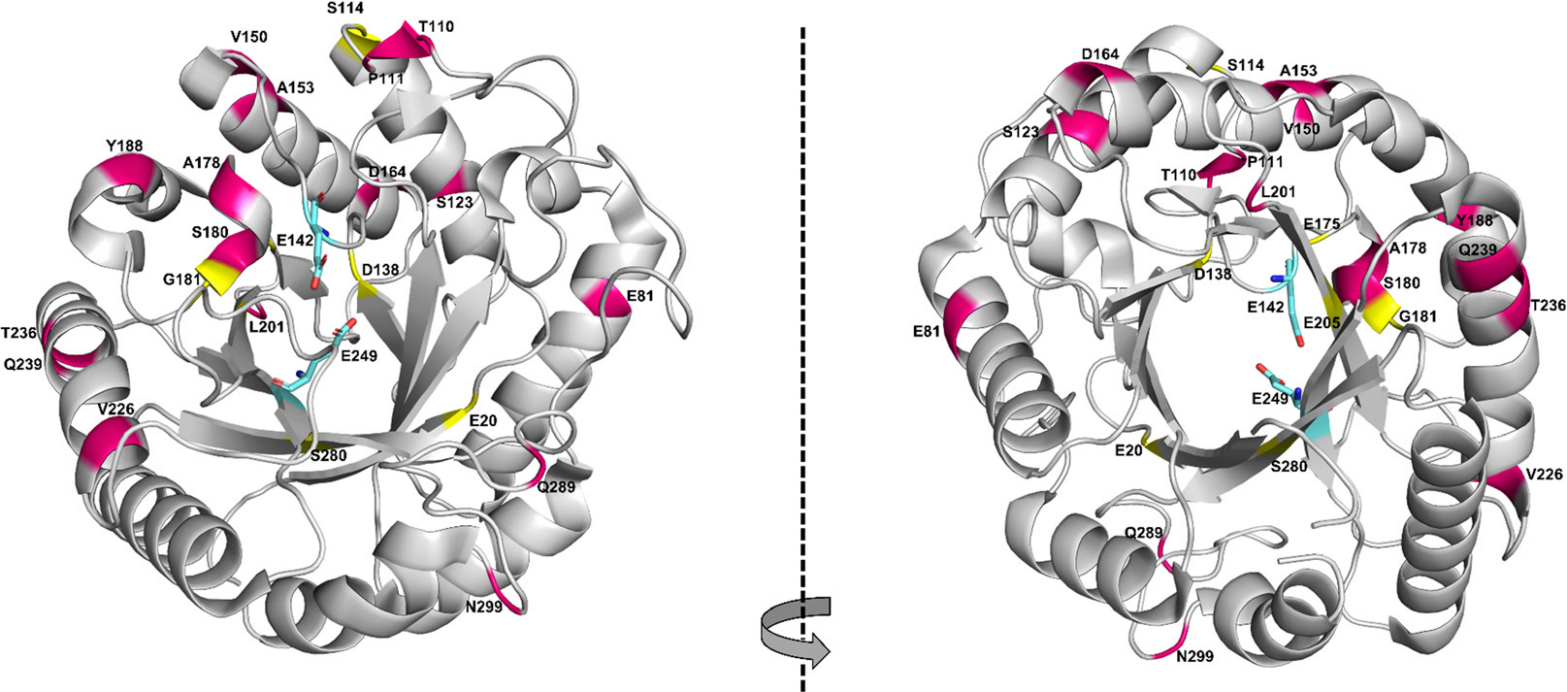
Positions of the top 20 EGLII variants with a single substitution identified by FoldX (yellow) and EVmutation (red). EVmutation identifies more distinct positions scattered across the enzyme, while positions identified by FoldX are more clustered, predominantly in the inner β-barrel region. The enzyme structure corresponds to PDB ID 5L9C, with the catalytic residues E142 and E249 shown in stick configuration.

For both methods, FoldX and EVmutation, the top 20 ranked variants with a single substitution were experimentally generated, expressed, and characterized with respect to thermal resistance and enzyme activity. Comparing the sets of the top 20 ranked variants with a single substitution from each method, we observed several key differences: (a) there was no overlap between the sets, likely due to the distinct selection filters applied by each method; (b) a significant disparity in the diversity of substituted positions emerged, with EVmutation, covering 16 distinct positions throughout the enzyme's structure, while the FoldX set contained only 7 unique positions, primarily within the inner β-barrel region (Fig. 1); (c) the top ranked substitutions from FoldX concentrated mainly on the β-sheets, whereas EVmutation's top ranked substitution focused on the α-helices; (d) conservation analysis using ConSurf^48^ revealed that FoldX primarily targeted conserved residues (4 highly conserved, 3 moderately conserved), whereas EVmutation favoured less conserved residues (1 highly, 6 moderately, and 9 poorly conserved) (Table S8 and Fig. S36 in ESI†).

These findings suggest that EVmutation explores a broader and more evolutionarily flexible sequence space, potentially identifying substitutions that enhance functional properties without compromising structural integrity.

### Enzymatic activity of EGLII variants after thermal stress

EGLII variants, including the 20 with the lowest ΔΔ*G* values and the 20 with the highest Δ*E* values (Table 1), along with the wild type, were subjected to thermal stress by incubation at 75 °C for 60 minutes.

**Fig. 2:**
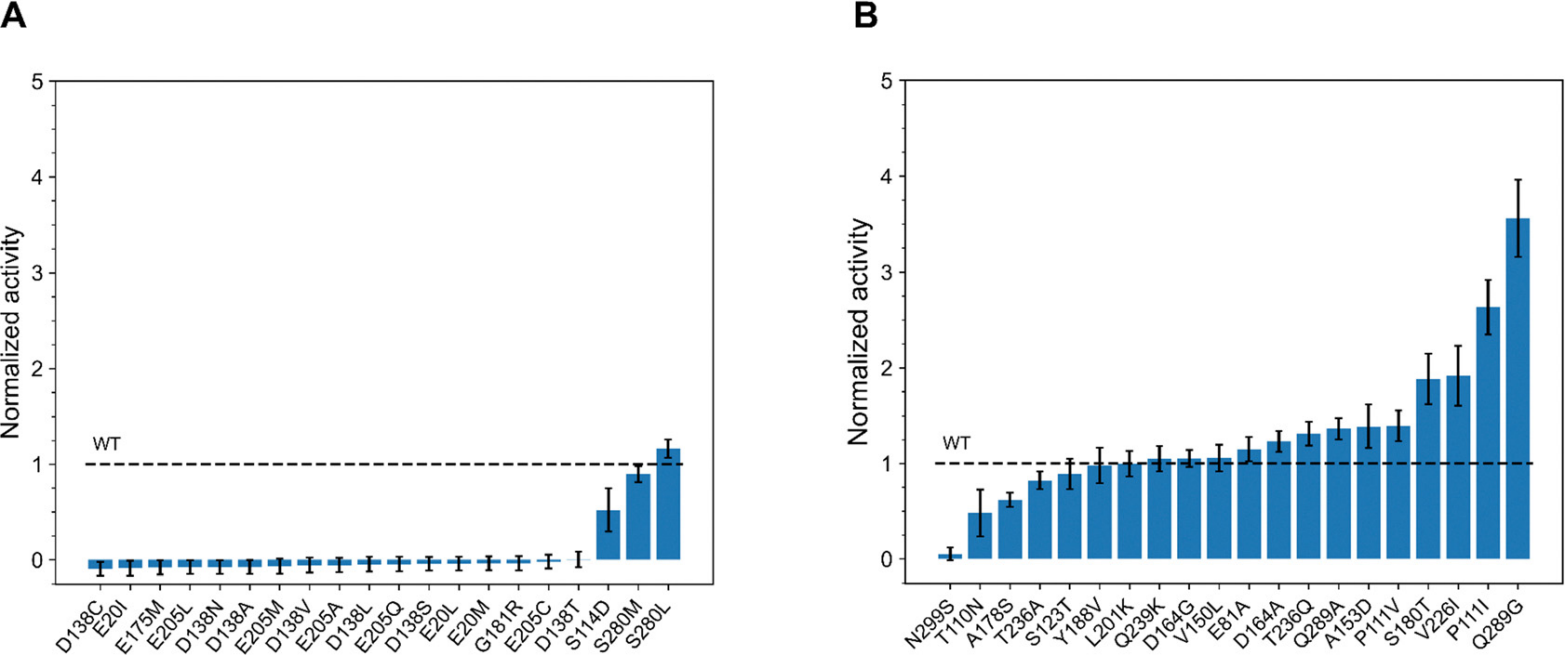
Normalized enzymatic activity of predicted single-substituted variants relative to the WT (dashed line) after 60 minutes incubation at 75 °C. (A) Top 20 variants predicted by FoldX. Most variants showed reduced enzymatic activity, with S208L demonstrating the highest improvement with (1.2 ± 0.1)-fold. (B) Top 20 variants predicted by EVmutation. Most variants exhibited enhanced enzymatic activity, with Q289G showing the highest improvement at (3.6 ± 0.4)-fold. The error bars represent the SEM calculated from at least three independent biological replicates.

Following incubation, their enzymatic activities were measured using a hydrolytic activity assay (see Experimental: Enzymatic assay). Fig. 2 displays the normalized enzymatic activities of the variants relative to the EGLII wild type (WT).

Among the FoldX variants, one variant showed increased activity, one showed similar activity, while the remaining 18 variants exhibited either reduced activity or were inactive compared to the wild type (Fig. 2A). The most active variant, S280L, demonstrated a (1.2 ± 0.1)-fold improvement in activity compared to the WT. Without thermal stress prior to the activity assay, most variants exhibited either reduced or similar activity compared to the WT (Fig. S18 in ESI†).

We applied a similar procedure to assess the activity of the EVmutation variants. After 60 minutes of incubation at 75 °C, 10 variants displayed increased activity, 6 showed similar activity, and 3 showed reduced activity compared to the WT (Fig. 2B). Among the beneficial variants, Q289G, P111I, V226I, and S180T demonstrated significant improvements in enzymatic activity, ranging from (1.9 ± 0.3) to (3.6 ± 0.4)-fold. Conversely, T110N, A178S, and N299S exhibited notably reduced activity. Without thermal stress, 9 variants were more active than the WT, 10 showed similar activity, and only one variant showed reduced activity (Fig. S19 in ESI†).

In summary, among the 20 FoldX variants with the lowest ΔΔ*G* values, increased activity after thermal stress was observed in only one variant. In contrast, 10 out of the top 20 EVmutation variants, ranked by Δ*E* values, demonstrated improved activity after thermal stress. This analysis demonstrates that using statistical energy (Δ*E*) to identify EGLII variants with a single substitution for increased activity at elevated temperatures is more effective than using free energy differences (ΔΔ*G*).

### Thermodynamic stability of EGLII variants

In addition to measuring the enzymatic activity of the EGLII variants, we also assessed their thermodynamic stability by determining melting temperatures (*T*_m_) using nanoDSF (Table 2). Measurements were conducted across a temperature range of 20–95 °C, with an increment of 1 °C per minute (thermal unfolding curves are shown in Fig. S24–S35 in ESI†).

**Table 2:** Melting temperatures (*T*_m_) of EGLII wild type and single-substituted variants predicted by FoldX and EVmutation. Values are expressed as the mean ± SEM, based on at least two independent biological replicates

FoldX		EVmutation	
Variant	*T*_m_ (°C)	Variant	*T*_m_ (°C)
G181R	84.8 ± 0.1	Q289G	85.0 ± 0.2
S280L	84.2 ± 0.2	P111I	83.5 ± 0.3
S280M	84.1 ± 0.2	S180T	83.2 ± 0.2
S114D	82.6 ± 0.1	V226I	83.1 ± 0.2
WT	82.2 ± 0.1	Q289A	82.8 ± 0.2
E205L	80.9 ± 0.1	T236Q	82.8 ± 0.2
E205C	79.5 ± 0.1	S123T	82.8 ± 0.4
E205Q	78.8 ± 0.2	V150L	82.8 ± 0.2
E205M	78.5 ± 0.2	A153D	82.8 ± 0.3
E205A	78.4 ± 0.2	T236A	82.7 ± 0.3
D138A	74.5 ± 0.1	Q239K	82.6 ± 0.3
D138S	74.4 ± 0.1	P111V	82.5 ± 0.2
D138N	74.4 ± 0.4	D164G	82.4 ± 0.3
D138T	73.8 ± 0.3	L201K	82.3 ± 0.2
D138L	73.3 ± 0.2	E81A	82.3 ± 0.3
D138C	73.2 ± 0.1	WT	82.2 ± 0.1
D138V	73.1 ± 0.2	D164A	82.1 ± 0.0
E20I	72.6 ± 0.1	T110N	81.7 ± 0.1
E20L	70.9 ± 0.1	Y188V	81.7 ± 0.3
E20M	70.2 ± 0.1	A178S	81.0 ± 0.2
E175M	55.3 ± 1.4	N299S	80.8 ± 0.1

Variants predicted by EVmutation demonstrated better thermodynamic stability compared to those predicted by FoldX, both in terms of number and their *T*_m_ values (Table 2). As EGLII is already a thermostable enzyme with an observed *T*_m_ of (82.2 ± 0.2) °C, improvements in *T*_m_ were modest but notable, reaching a maximum of (85.0 ± 0.2) °C for the Q289G variant. Among the EGLII variants predicted by FoldX, 3 displayed higher melting temperatures than the WT. Notably, G181R exhibited an increased *T*_m_ of (84.8 ± 0.1) °C, though this variant was enzymatically inactive (Fig. 2A). Variants S280L and S280M, which retained enzymatic activity, showed Δ*T*_m_ values of (2.0 ± 0.3) °C and (1.9 ± 0.3) °C, respectively. While S114D had a *T*_m_ comparable to the WT, the remaining 16 variants demonstrated significant decreases in *T*_m_, with reductions reaching up to Δ*T*_m_ = (−26.9 ± 1.5) °C (Table 2). For EVmutation's top-ranked variants, 12 out of 20 variants exhibited similar or slightly increased melting temperatures compared to WT. The remaining were evenly divided with 4 variants demonstrating significantly increased melting temperatures and 4 showing reduced *T*_m_ values than WT. Notably, the variant Q289G, which displayed the highest enzymatic activity after exposure to thermal stress (Fig. 2B), showed the most significant increase in melting temperature, with Δ*T*_m_ = (2.8 ± 0.3) °C (Table 2). This was followed by P111I, S180T, and V226I, all of which also maintained catalytic activity after exposure to elevated temperature. A positive correlation between the melting temperature and enzymatic activity was observed for the EVmutation-predicted EGLII variants, suggesting that EVmutation may effectively mediate the activity-stability tradeoff, potentially optimizing stability without compromising enzyme function.

### Half-lives of EGLII variants

**Fig. 3:**
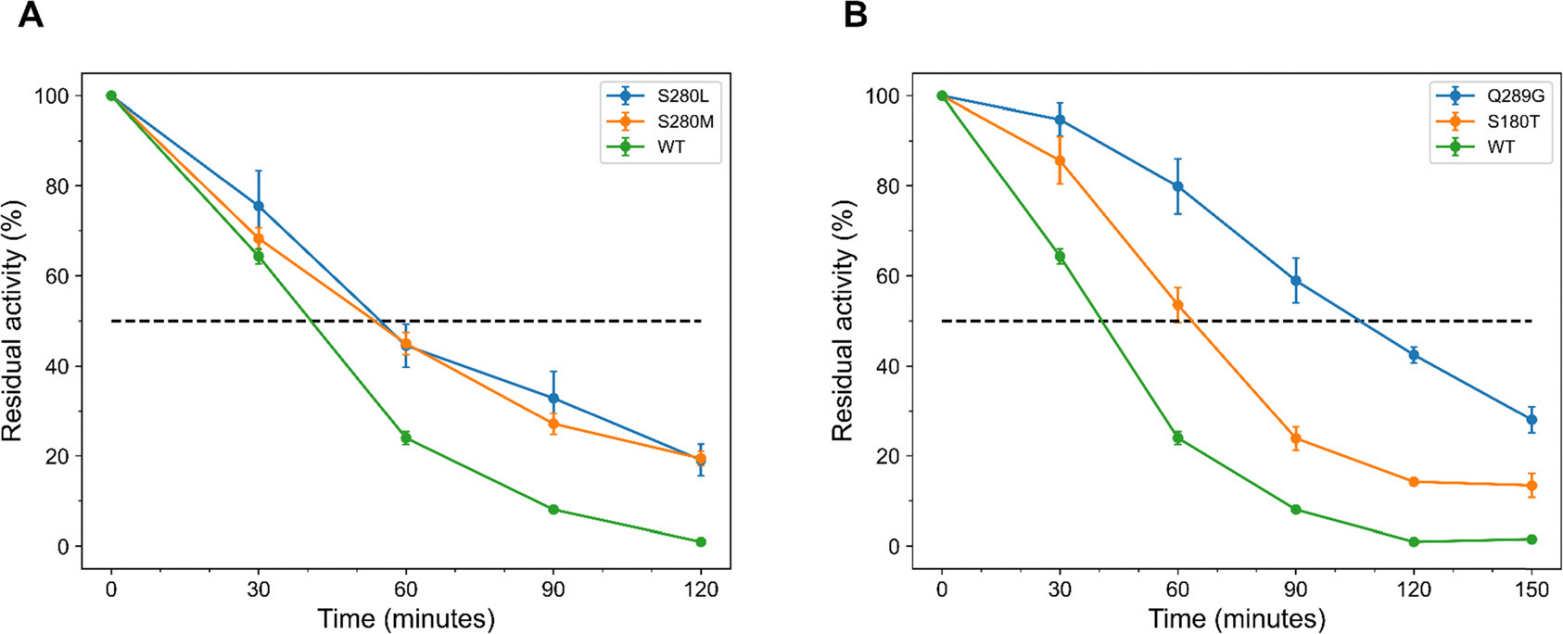
EGLII variants with a single substitution and extended half-lives at 75 °C, selected from the top 20 ranked by FoldX and EVmutation. (A) FoldX variants with the longest half-lives. S280M and S280L, displaying half-lives of (54 ± 8) and (54 ± 2) minutes, compared to the WT half-life of (40 ± 2) minutes. (B) EVmutation variants with the longest half-lives. Q289G and S180T, showing significantly enhanced half-lives of (104 ± 7) and (63 ± 4) minutes, respectively. The error bars represent the SEM from at least three independent biological replicates.

The kinetic stability of the predicted EGLII variants (Table 1) was assessed by determining their half-lives at 75 °C. Among the top 20 variants predicted by FoldX, only two exhibited increased half-lives compared to the WT, which has a half-life of (40 ± 2) minutes (Table S7 and Fig. S15 in ESI†). Specifically, S280L and S280M demonstrated extended half-lives of (54 ± 8) and (54 ± 2) minutes (Fig. 3A). In contrast, the top 20 variants predicted using EVmutation showed more consistent improvements in half-lives, with 15 out of 20 variants exceeding the WT's half-life (Table S7 and Fig. S16 in ESI†). The most notable enhancement was observed for the variant Q289G, which achieved a half-life of (104 ± 7) minutes, followed by S180T and V226I, with half-lives of (63 ± 4) and (60 ± 7) minutes (Fig. 3B).

It was observed that EVmutation outperformed FoldX in predicting EGLII variants with increased half-lives. This method not only identified a greater number of variants with increased melting temperature (Table 2) but also yielded variants with significantly improved half-lives, with 75% of the tested variants surpassing the WT's half-life (Table S7 in ESI†).

### Beyond variants with a single substitution

The experimental characterization of the top 20 ranked single-substituted EGLII variants, predicted by FoldX and EVmutation, demonstrated that Δ*E* is a more reliable measure than ΔΔ*G* for identifying variants with improved activity and thermal resistance. Motivated by these findings, we evaluated EVmutation's capability to predict improved variants with multiple substitutions. Consequently, EVmutation was employed to predict Δ*E* for EGLII variants with two substitutions. Similarly, the 20 candidates with the highest Δ*E* values were generated, expressed, and characterized for enzymatic activity and thermal resistance. Notably, a general trend emerged: most double-substituted variants included at least one top-ranked single substitution (Tables 1 and 3).

**Table 3:** Changes in statistical energy (Δ*E*) and melting temperatures (*T*_m_) of the 20 double-substituted EGLII variants with the highest Δ*E* predicted by EVmutation. The Q289G variant and the wild type (WT) values are given as reference. *T*_m_ values are expressed as the mean ± SEM, based on at least two independent biological replicates

Variant	Δ*E*	*T*_m_ (°C)
S180T/Q289G	4.43	85.9 ± 0.1
P111I/Q289G	5.47	85.7 ± 0.3
P111V/Q289G	4.81	85.6 ± 0.2
V226I/Q289G	5.83	85.4 ± 0.3
T236Q/Q289G	4.10	85.3 ± 0.2
T110N/Q289G	4.44	85.2 ± 0.1
D164A/Q289G	5.41	85.1 ± 0.3
D164G/Q289G	4.09	85.1 ± 0.3
E81A/Q289G	4.19	85.0 ± 0.2
**Q289G**	**3.13**	**85.0 ± 0.2**
Q289G/N299S	4.35	84.3 ± 0.2
P111V/V226I	4.26	83.8 ± 0.1
A178S/Q289G	5.23	83.8 ± 0.2
P111I/V226I	5.12	83.7 ± 0.3
P111I/Q289A	4.15	83.4 ± 0.3
P111I/D164A	4.85	83.2 ± 0.3
V226I/Q289A	4.37	83.0 ± 0.2
D164A/V226I	5.03	82.8 ± 0.3
P111I/A178S	4.49	82.6 ± 0.2
**WT**	**0.00**	**82.2 ± 0.1**
A178S/V226I	4.55	81.7 ± 0.2
D164A/A178S	4.31	81.2 ± 0.1

**Fig. 4:**
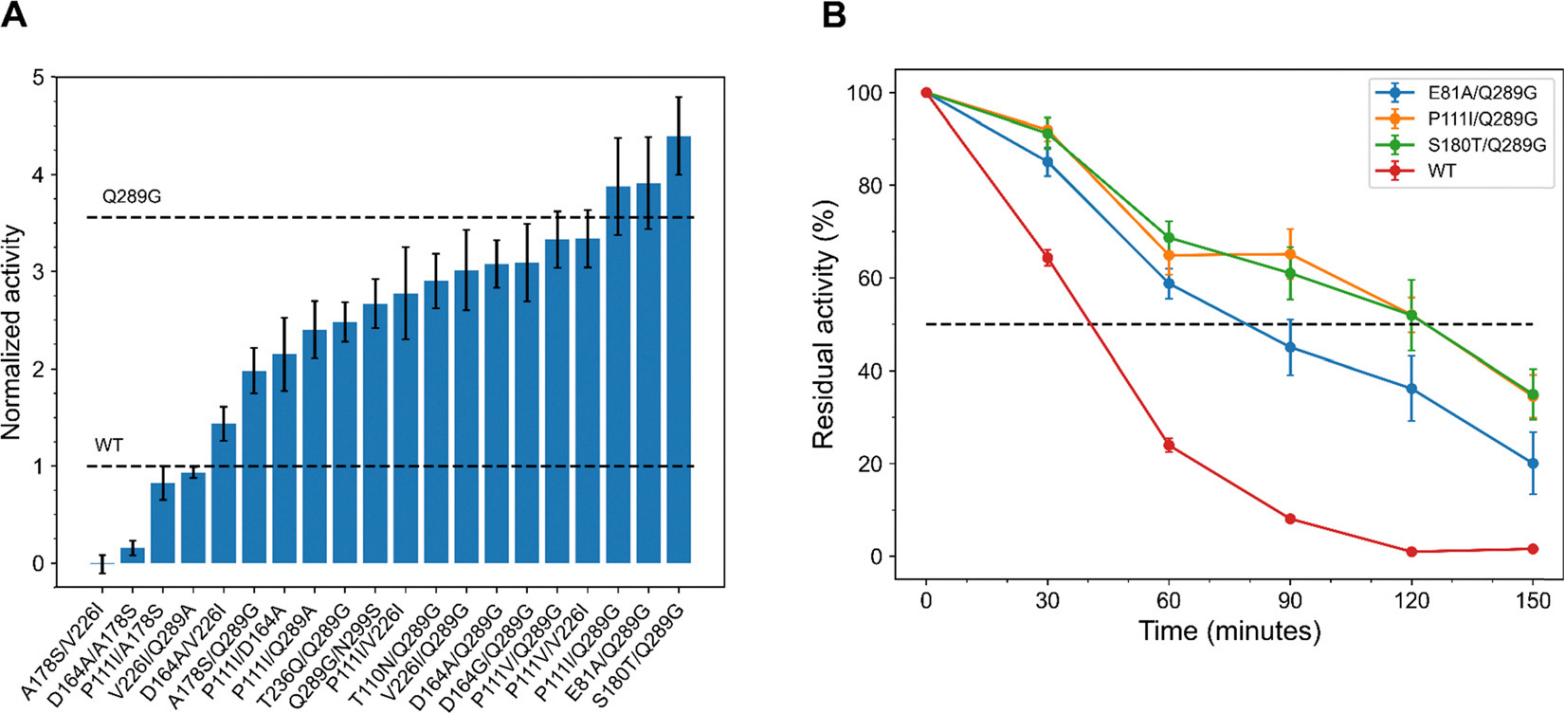
Normalized enzymatic activity and half-lives of the top 20 double-substituted EGLII variants predicted by EVmutation. (A) Normalized enzymatic activity of the predicted EGLII variants relative to the WT after 60 minutes of incubation at 75 °C. Nearly 80% of the variants exhibited enhanced enzymatic activity, with the S180T/Q289G variant showing the greatest improvement, achieving a (4.4 ± 0.4)-fold increase. Three variants also demonstrated higher improvement than the best single-substituted variant predicted by EVmutation (Q289G). (B) Half-lives of the most improved double-substituted EGLII variants at 75 °C, as predicted by EVmutation. The three most improved variants, P111I/Q289G, S180T/Q289G, and P111V/Q289G, demonstrated substantial kinetic stability, with half-lives of (122 ± 8), (121 ± 15), and (118 ± 7) minutes.

The double-substituted variants were subjected to thermal stress by incubation at 75 °C for 60 minutes. Among the 20 variants tested, 16 showed enhanced enzymatic activity, 1 had similar, and 3 were found to be less active compared to the WT (Fig. 4A). Notably, three variants demonstrated more than a threefold improvement compared to the WT with (3.9 ± 0.5)-fold for E81A/Q289G and P111I/Q289G, and (4.4 ± 0.4)-fold for S180T/Q289G. These double-substituted variants surpassed the highest-performing single variant Q289G, which showed an activity improvement of (3.6 ± 0.4)-fold compared to the WT under similar conditions. In the absence of thermal stress, 13 out of the 20 variants demonstrated higher activity than the WT enzyme, while 4 showed similar activity and 2 exhibited reduced activity (Fig. S20 in ESI†). Interestingly, D164G/Q289G and T236Q/Q289G, which did not show improved activity in the absence of thermal stress, displayed significantly enhanced activity after incubation at 75 °C, with improvements of (3.1 ± 0.4)-fold and (2.5 ± 0.2)-fold, respectively. We also observed that many improved variants with two substitutions at elevated temperatures included Q289G as one of the substitutions (11 out of 16), while the non-improved variants contained A178S.

The double-substituted variants also showed notably improved thermodynamic stability. There was an increase in the number of variants with enhanced melting temperatures, with over 6 displaying a Δ*T*_m_ of more than 3 °C (Table 3). The S180T/Q289G variant showed the greatest improvement, with a Δ*T*_m_ of (3.7 ± 0.2) °C, followed by P111I/Q289G (3.5 ± 0.3) °C, P111V/Q289G (3.4 ± 0.3) °C, V226I/Q289G (3.2 ± 0.4) °C, T236Q/Q289G (3.1 ± 0.2) °C, and T110N/Q289G (3.1 ± 0.2) °C. Upon evaluation of the kinetic stability of the 20 variants at 75 °C, 15 showed increased half-lives, 2 exhibited half-lives comparable to the WT, and 3 had reduced half-lives (Table S7 and Fig. S17 in ESI†). Notably, the P111I/Q289G, S180T/Q289G, and P111V/Q289G variants demonstrated the most substantial improvements, with half-lives of (122 ± 8), (121 ± 15), and (118 ± 7) minutes (Fig. 4B). We observed that many improved variants with two substitutions include the Q289G substitution, whereas non-improved variants often included A178S. Interestingly, the half-life of the single variant Q289G (104 ± 7) minutes and activity was higher than that of most variants with two substitutions containing this substitution. This suggests that combining Q289G with other substitutions may lead to a decrease in half-life, potentially due to underlying epistatic interactions.

Our analysis revealed that selecting variants with two substitutions based on Δ*E* is an effective strategy for identifying improved variants. Despite a bias towards the Q289G substitution, we observed double-substituted variants demonstrating higher enzymatic activities after thermal stress, increased melting temperatures, and extended half-lives compared to the best-performing single-substitution variant. This suggests that EVmutation is capable of zero-shot predictions for variants containing multiple substitutions, allowing engineering campaigns to start directly with these variants. This can further reduce the number of rounds and time required to enhance the enzyme's properties.

### Discussion

**Fig. 5:**
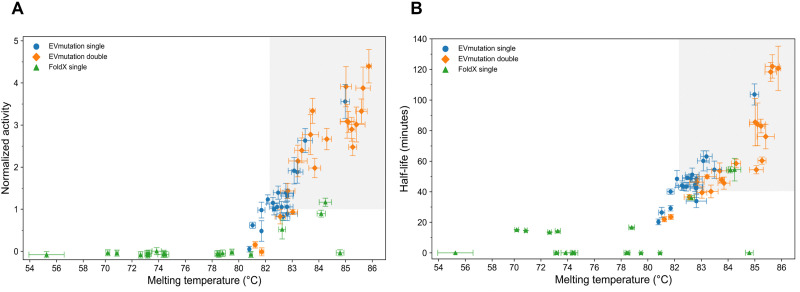
Correlation between thermal resistance and enzymatic activity of EGLII variants predicted by FoldX and EVmutation (single- and double-substituted). The grey shaded regions highlight areas with higher thermal resistance and enzymatic activity. (A) Correlation between normalized activity at 75 °C and melting temperature (*T*_m_). EVmutation predictions show a stronger correlation than FoldX predictions, with EVmutation double-substituted variants outperforming both EVmutation single-substituted and FoldX variants. (B) Correlation between half-life at 75 °C and melting temperature (*T*_m_). Again, EVmutation predictions demonstrate a stronger correlation than FoldX predictions, with EVmutation double-substituted variants consistently outperforming both EVmutation single-substituted and FoldX variants.

In this study, we assessed the potential of FoldX's ΔΔ*G* and EVmutation's Δ*E* as filters for identifying thermally resistant and active EGLII variants. Our results with the top 20 ranked variants show that 50% of the variants with a single substitution selected using Δ*E* exhibited enhanced enzymatic activity after thermal stress, compared to only one variant identified using ΔΔ*G* as the ranking criterion (Fig. 2). Beyond improvements in activity, both methods effectively identified variants with higher melting temperatures and extended half-lives, with the EVmutation-based approach demonstrating greater efficacy (Table 2 and Fig. 5).

Computational methods can significantly reduce the screening effort required to identify improved enzyme variants, especially when compared to traditional approaches such as random mutagenesis *via* error-prone PCR (epPCR). For instance, epPCR previously yielded only 22 variants with increased activity out of approximately 8000 EGLII variants (hit rate of 0.27%),^31^ highlighting the efficiency achieved with ΔΔ*G* and Δ*E* as selection filters. Although FoldX has been successfully employed in various studies to enhance enzyme thermal resistance, in our case, its predicted ΔΔ*G* values proved to be a less accurate selection filter. Several possible reasons could explain this outcome. First, the accuracy of FoldX predictions heavily depends on the quality of the input protein structure. Although we used a crystal structure with reasonably high resolution (PDB ID 5L9C; resolution 1.8 Å^49^), even minor inaccuracies or missing structural details—such as unresolved loops, flexible regions, or alternate conformations—can introduce errors in energy calculations. Notably, the first ten N-terminal residues were missing in this structure, which may have influenced predicted stability if that region contributes to early folding events or forms stabilizing interactions within the native structure. Additionally, FoldX uses an empirical energy function that calculates the free energy change (ΔΔ*G*) upon point mutation. This involves computing an effective energy score based on a single, static protein conformation, incorporating simplified models of van der Waals interactions, hydrogen bonding, solvation, and entropic contributions. While this approach enables rapid and scalable predictions, making it computationally efficient, it does not simulate physical movements or account for protein dynamics, conformational flexibility, and long-range interactions—all of which are crucial for accurate stability predictions.^50^ This simplification can limit the predictive power of FoldX, especially for proteins where stability and function are tightly coupled to conformational dynamics. Moreover, FoldX is primarily designed to assess structural stability rather than functional effects, such as enzymatic activity, which may involve distant or subtle interactions not captured in its calculations. Interestingly, in our case, the predicted variants also exhibited lower thermodynamic stability, with 16 out of 20 EGLII variants showing reduced thermodynamic stability (Table 2). These results are consistent with a report by Buss *et al.*, suggesting that FoldX may be more accurate at predicting destabilizing mutations than stabilizing ones.^46^

In contrast, EVmutation leverages statistical couplings derived from multiple sequence alignments, capturing evolutionary constraints that implicitly include functional and structural considerations. In our study, EVmutation's Δ*E* proved highly effective at identifying improved EGLII variants with both single and double substitutions. The predictive hit-rate increased from 50% for single substitutions to 80% for double substitutions, indicating strong predictive performance even for more complex combinatorial changes. We further tested EVmutation's Δ*E* by engineering another enzyme, *Bacillus subtilis* lipase A (BSLA). We observed substantial improvements in thermal resistance and activity (65% hit-rate, up to 5.4-fold half-life improvement; Table S5 and Fig. S3, S4 in ESI†), thus highlighting the broader applicability of EVmutation across enzymes with varying intrinsic stabilities.

In addition to its predictive accuracy, EVmutation offers a significant advantage in computational efficiency. The method is much faster and enables substantial *in silico* throughput, requiring only summation of matrix elements once the parameters of the statistical model are inferred. This makes EVmutation particularly advantageous for exploring larger portions of the protein space. In contrast, FoldX requires significantly more computational resources to evaluate the energy function, limiting its suitability for exploring the protein space due to the need for new calculations with each prediction.

Our results also suggested that EVmutation successfully mediates the activity-stability tradeoff, as structurally stable variants exhibited both enhanced enzymatic activity and extended half-lives. To investigate how the predicted substitutions influenced both thermal resistance and enzymatic performance, we used Spearman's rank correlation coefficient (*ρ*) to analyse the relationships between melting temperature (*T*_m_), normalized enzymatic activity (Fig. 5A), and half-life (Fig. 5B) under thermal stress. Among single-substituted variants, those predicted by EVmutation showed strong positive correlations between *T*_m_ and both normalized activity (*ρ* = 0.77, *p* = 6.1 × 10^−5^) and half-life (*ρ* = 0.69, *p* = 7.6 × 10^−4^). In comparison, EGLII variants predicted by FoldX showed weaker correlations (*ρ* = 0.77 for activity; and *ρ* = 0.18 for half-life). These findings indicate that EVmutation can successfully identify individual mutations that enhance both stability and function. For double-substituted variants, correlations were even stronger: the correlation between *T*_m_ and activity increased to 0.83 (*p* = 6.4 × 10^−6^), and for half-life it reached 0.93 (*p* = 4.6 × 10^−9^). Moreover, as shown in Fig. 5, most of the EVmutation-predicted variants clustered in the high-*T*_m_/high-activity region (grey shaded area), suggesting that the method can enhance both thermal resistance and enzymatic performance.

While EVmutation demonstrated strong predictive performance, it also has notable limitations. First, neither the predicted statistical energy (Δ*E*) nor the predicted free energy of unfolding (ΔΔ*G*) showed strong direct correlations with experimentally measured melting temperatures (*T*_m_) and normalized enzymatic activities (Fig. S5 and Table S6 in ESI†), highlighting the constraints of zero-shot prediction accuracy. Additionally, EVmutation displayed limited diversity among the top-ranked double-substitution variants, predominantly favouring combinations that included the highest-ranked single substitution (Q289G), suggesting a bias in the predictive model. While most of the double-substituted variants outperformed the wild type enzyme, several combinations containing Q289G did not exhibit additive or synergistic improvements. Only one variant P111V/V226I showed additive effect while 19 out of 20 variants showed negative epistatic interactions. Such non-additive effects likely arise from structural or functional conflicts between substitutions, an aspect not explicitly modeled by EVmutation's statistical framework. Furthermore, EVmutation uses evolutionarily related sequences of the enzyme to infer model parameters, Δ*E*-based selection may be less effective for engineering non-natural properties such as stability in organic solvents or ionic liquids as such information is not available in the sequence alignment. Finally, the quality of this statistical model depends on the number of available homologous sequences; therefore, it may yield suboptimal results for enzymes with fewer homologs.

Addressing these limitations could further enhance EVmutation's predictive accuracy, variant diversity, and overall applicability. First, fine-tuning internal model parameters (sequence weighting and pseudo-count regularization) may help mitigate inherent biases toward certain substitutions, encouraging the exploration of more diverse mutational combinations. Additionally, incorporating task-specific evolutionary constraints—such as penalizing overrepresented residues or promoting diversity in targeted regions—could further expand the exploration of sequence space. These enhancements might reduce reliance on a single dominant substitution, thereby increasing the likelihood of identifying synergistic combinations. Furthermore, incorporating epistasis-aware modelling through supervised machine learning algorithms trained on comprehensive experimental datasets could significantly improve predictions of combinatorial variants, increasing the likelihood of identifying synergistic substitutions. Finally, integrating EVmutation with complementary computational methods, such as molecular dynamics (MD) simulations, could further enrich variant selection by explicitly accounting for structural dynamics, flexibility, and solvent interactions, expanding its predictive scope to non-natural enzyme properties.

Despite its limitations, EVmutation remains highly an efficient, unsupervised, zero-shot approach for identifying beneficial variants based solely on sequence data. Although Δ*E* values did not quantitatively correlate with experimental *T*_m_ or activity, EVmutation-predicted variants preferentially clustered in regions of higher *T*_m_ and activity compared to FoldX predictions (Fig. S5 and Table S6 in ESI†). While some double-substituted variants underperformed relative to Q289G alone, many exceeded the performance of the wild type. Our findings indicate that EVmutation effectively identifies beneficial enzyme variants while efficiently mediating the trade-off between stability and activity. This highlights its practical utility as an initial screening tool in protein engineering campaigns especially when experimental throughput or structural information is limited.

To our knowledge, this is the first study employing EVmutation to improve the thermal resistance and activity of cellulases. Owing to its simplicity, effectiveness, and computational efficiency, EVmutation represents a promising method for *a priori* identification of beneficial variants in protein engineering campaigns. With further methodological refinements, its predictive accuracy and applicability are expected to improve, ultimately minimizing experimental efforts and accelerating protein engineering.

## Conclusion

The challenge of simultaneously enhancing enzyme activity and thermal resistance is complex, but this study demonstrated that EVmutation can efficiently address it. Specifically, EVmutation predicted beneficial variants with enhanced thermal resistance and enzymatic activity, even for double-substituted variants. The simplicity, effectiveness, and computational efficiency of EVmutation makes it a valuable tool for experimentalists to enhance enzyme properties with minimal experimental efforts. In protein engineering campaigns, EVmutation could provide the *a priori* identification of beneficial substitutions, reducing the time and number of iterative rounds required in traditional directed evolution methods like standard epPCR. Furthermore, EVmutation is likely applicable for many enzymes to enhance their properties if an extensive evolutionary sequence dataset is available.

## Experimental

### Chemicals and reagents

All commercially available chemicals were purchased at analytical or higher grade from Merck (Darmstadt, Germany), NewEngland Biolabs (Ipswich, USA), Applichem (Darmstadt, Germany), Carl Roth (Karlsruhe, Germany), unless otherwise specified. VeriFi™ polymerase mix was purchased from PCR Biosystems Ltd (London, UK). NucleoSpin plasmid kit from Micherey-Nagel GmbH & Co. KG (Düren, Germany) was used to isolate the plasmid DNA. All oligonucleotides were purchased salt-free from Eurofins Genomics.

### Strains and plasmid

*Escherichia coli* DH5α (Agilent Technologies, Santa Clara, USA) was used as the cloning host and *Pichia pastoris* BSYBG11 (Bisy e.U., Hofstaetten/Raab, Austria) was used as the expression host. The shuttle vector pBSYA1S1Z (Bisy e.U., Hofstaetten/Raab, Austria) with episomal expression and zeocin resistance was used. The codon optimized endo-β-Glucanase gene *eglII* from *Penicillium verruculosum* (UniProtKB: A0A1U7Q1U3) was cloned into pBSYA1S1Z as reported earlier^31^ (Fig. S1 in ESI†). The plasmid construct was then used to generate the 60 predicted EGLII variants using site-directed mutagenesis^51^ (see primer sequences in Table S2 in ESI†).

### Cell culture and expression

EGLII variants were cultured and expressed in 96-well F-bottom polystyrene microtiter plates (MTPs) (Greiner, Frickenhausen, Germany) using an MTP shaker (Infors HT Multitron, Bottmingen, Switzerland). YPD media (1% w/v yeast extract, 2% w/v peptone, and 2% w/v d-glucose) supplemented with 100 μg mL^−1^ Zeocin was used for cell culture and expression. Colonies picked from YPD agar plates were used to inoculate preculture (150 μL per well, 30 °C, 900 rpm, 48 hours, and 70% humidity). The main expression culture (150 μL per well, 25 °C, 900 rpm, 96 hours, and 70% humidity) was inoculated with a volume of 5 μL from the preculture. The crude enzyme was separated from the cell culture by centrifugation (Eppendorf 5810R; 4 °C, 3220×*g*, 15 minutes), and the endoglucanase containing cell culture supernatant was used for subsequent analysis.

### Enzymatic assay

To assess the hydrolytic activity of EGLII, solubilized Azo-CM-Cellulose (Megazyme, Bray, Ireland) was used as the substrate, following modified manufacturer instructions. The assay is based on the enzymatic hydrolysis of high-molecular-weight cellulose chains into low-molecular-weight cellulose. After cultivation in a 96-well MTP, EGLII containing supernatant was diluted fivefold with sodium acetate buffer (0.1 M, pH 4.5). This diluted supernatant was then incubated at high temperature (75 °C) for 60 minutes without the substrate. The temperature of 75 °C was chosen as it reduced the EGLII wild type enzyme activity to approximately 25%. Samples of the diluted supernatant with (*T*_60_) and without (*T*_0_) incubation were transferred into another MTP for activity measurement (40 μL). To initiate the enzymatic reaction, 40 μL of 2% Azo-CM-Cellulose in sodium acetate buffer (0.1 M, pH 4.5) was added to each well. The reaction mixture was incubated for 10 minutes at 50 °C with shaking (900 rpm) using an ELMI Ltd. SkyLine DTS-4 Digital Thermo Shaker (Riga, Latvia). The reaction was halted by adding a precipitating solution (40 g L^−1^ sodium acetate, 4 g L^−1^ zinc chloride, 80% ethanol). After centrifuging the precipitated mixture at 1000×*g* for 10 minutes, we transferred 100 μL of the clear supernatant to a new 96-well microtiter plate (MTP). We then measured the absorbance at 590 nm using CLARIOstar Plus microplate reader from BMG Labtech (Ortenberg, Germany) in spiral scan mode with 4 mm diameter and 50 flashes per well. The absorbance values represent the hydrolytic efficiency of the enzyme variants, and the assay was performed with at least three biological replicates.

### Thermodynamic and kinetic stability analysis

NanoDSF, a nano-format of differential scanning fluorimetry, was used to analyze the melting temperature (*T*_m_). EGLII variants and the empty vector were cultured in test tubes (25 °C, 96 hours, 220 rpm) using an Infors HT Minitron shaker (Bottmingen, Switzerland). Following cultivation, the supernatant was separated by centrifugation (Eppendorf 5810R; 4 °C, 3220×*g*, 15 minutes) and subsequently concentrated using Amicon Ultra-4 centrifugal filters (MWCO-10kDa). Buffer exchange was performed using sodium acetate buffer (0.1 M, pH 4.5) and the concentrated samples were then subjected to nanoDSF measurement using Prometheus NT.48 from NanoTemper Technologies GmbH (Munich, Germany). The excitation power was set to 10% and the measurement was done over a temperature range of 20–95 °C, with temperature gradient of 1 °C minute^−1^. All measurements were performed with at least two replicates.

To determine the kinetic stability, the half-lives of EGLII variants at 75 °C was measured. EGLII variants and empty vector were cultured in 96-well F-bottom polystyrene MTPs (25 °C, 900 rpm, 96 hours, and 70% humidity). The cell culture supernatant was separated by centrifugation (Eppendorf 5810R; 4 °C, 3220×*g*, 15 minutes). The supernatant containing endoglucanase was incubated at 75 °C for different time intervals (0–150 minutes). Subsequently, the hydrolytic assay using Azo-CM-Cellulose was performed with the incubated and non-incubated supernatant as described above. All measurements were conducted with at least three biological replicates.

### FoldX

FoldX is a software suite capable of quantifying the energetic impacts of amino acid substitutions on the free energy of unfolding (Δ*G*) of a protein. Specifically, Δ*G* denotes the difference in free energy between the unfolded (*G*_unfolded_) and folded state (*G*_folded_) of the protein:

1 Δ*G* = *G*_unfolded_ − *G*_folded_

The free energy of unfolding is calculated using the following equation:^35^

2 Δ*G* = *a* Δ*G*_vdw_ + *b *Δ*G*_solvH_ + *c *Δ*G*_solvP_ + *d *Δ*G*_wb_ + *e *Δ*G*_hbond_ + *f *Δ*G*_el_ + *g *Δ*G*_kon_ + *h T*Δ*S*_mc_ + *i T*Δ*S*_sc_ + *j *Δ*G*_clash_

In this equation, the variables *a* to *j* represent the relative weights assigned to various energy terms. FoldX incorporates multiple energy terms, including solvent interactions, which are divided into hydrophobic (Δ*G*_solvH_) and polar (Δ*G*_solvP_) components. It also accounts for explicit water-mediated hydrogen bonds (Δ*G*_vb_), van der Waals forces (Δ*G*_vdw_), hydrogen bonding (Δ*G*_hbond_), and electrostatic interactions for monomers (Δ*G*_el_) as well as protein complexes (Δ*G*_kon_). Entropy calculations include determining *T*Δ*S*_sc_ for the side chains and *T*Δ*S*_mc_ for the backbone. Lastly, Δ*G*_clash_ accounts for steric overlaps between atoms in the structure.

FoldX estimates the energy difference between a wild type (Δ*G*_WT_) protein and its variant (Δ*G*_Variant_) by determining the change in free energy (ΔΔ*G*) between the unfolded and folded states of both structures:

3 ΔΔ*G* = Δ*G*_WT_ − Δ*G*_Variant_

First, the structure with PDB ID 5L9C^49^ was retrieved from the RCSB Protein Data Bank. Next, water molecules and heteroatoms were removed. Stability calculations were performed using FoldX (version 4.0) on a single monomer (chain A). The structure was relaxed, and hydrogen atoms were added using the ‘RepairPDB’ command. ΔΔ*G* values for all variants with a single substitution were calculated as the mean of five independent runs using the ‘BuildModel’ command. The variants were ranked based on their predicted ΔΔ*G*, and the top 20 most stable variants (negative ΔΔ*G*) were selected for experimental characterization.

### EVmutation

Direct coupling analysis is a statistical modeling technique used to reveal direct residue–residue interactions in proteins based on a multiple sequence alignment (MSA). It involves inferring the least-constrained model4
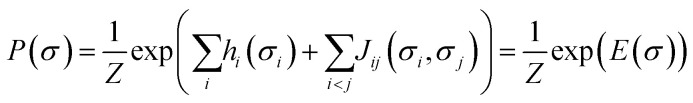
where *h*_*i*_(*σ*_*i*_) represents the local bias and *J*_*ij*_(*σ*_*i*_,*σ*_*j*_) is the coupling term. The model is constrained to reproduce the empirical occurrence frequencies (*f*_*i*_, *f*_*ij*_) observed in the MSA5
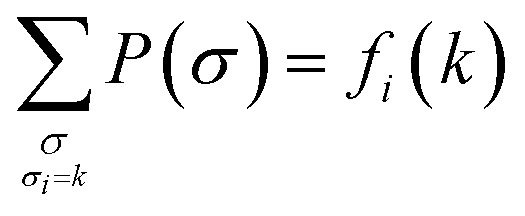
6
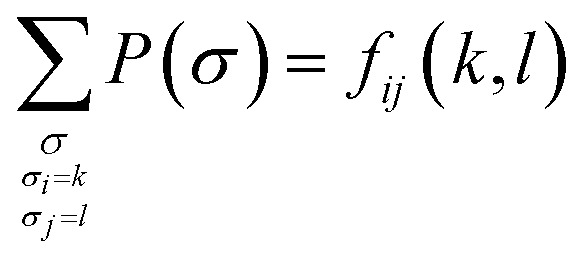
with *σ*_*i*_ denoting the amino acid at position *i* in the protein sequence *σ*, and *k*, *l* representing one of the twenty proteinogenic amino acids.^36,52,53^

DCA has been used to identify residues critical for protein–protein interactions and to assist in the structural determination of protein complexes.^54^ In 2015, Figliuzzi *et al.* introduced a difference score, calculated as follows:^55^

7 Δ*E*(*σ*,*σ*^wt^) = *E*(*σ*) − *E*(*σ*^wt^)

By demonstrating a correlation between Δ*E* and protein fitness across various enzymes and datasets, Hopf *et al.* validated the general applicability of their DCA-based method, EVmutation, for quantifying substitution effects.^36^ This allowed a wider audience, including protein engineers, to leverage the potential of this method for identifying beneficial substitutions based solely on sequence data. Given the high costs and labour-intense nature of structure determination and generating assay-based labelled data, a DCA-based approach offers significant advantages for protein engineering.^47^

An MSA was generated by performing a jackmmer search (HMMER 3.3.2)^56^ against the UniProt Reference Clusters (UniRef) database UniRef100 (release 2021_03).^57,58^ The search was initialized with the EGLII sequence using a bit score of half the sequence length. The MSA was then post-processed following the procedure outlined by Hopf *et al.*, removing sequences with more than 50% gaps and positions with gaps in over 30% of the sequences. Local biases and coupling terms were inferred using plmc (release 16 May 2018, available at https://github.com/debbiemarkslab/plmc) with regularization parameters *l*_h_ = 0.01 and *l*_e_ = 0.2 × (*N* − 1), where *N* = 294 is the number of effective sites in the alignment. Δ*E* was calculated for all variants with one and two substitutions.

## Author contributions

AK: conceptualization, data curation, formal analysis, investigation, methodology, software, visualization, writing – original draft, writing – review & editing. AI: conceptualization, data curation, formal analysis, investigation, methodology, software, visualization, writing – original draft, writing – review & editing. NG: investigation, methodology, software, discussion, writing – review & editing. FC: supervision, writing – review & editing. MD: resources, supervision. US: funding acquisition, resources, discussion, supervision, writing – review & editing.

## Data availability

The data supporting this article have been included as part of the ESI.† Additional raw data supporting the study's findings are available from the corresponding author upon reasonable request.

## Conflicts of interest

There are no conflicts to declare.

## Supplementary Material

CB-006-D5CB00013K-s001

CB-006-D5CB00013K-s002

CB-006-D5CB00013K-s003

CB-006-D5CB00013K-s004
