# Supplementary material for: Exploring sequence- and structure-based fitness landscapes to enhance thermal resistance and activity of endoglucanase II with minimal experimental effort
Source: RSC Chem Biol. 2025 May 5;6(6):975–86. doi: 10.1039/d5cb00013k (PMC12080268; doi:10.1039/d5cb00013k)
Supplement: CB-006-D5CB00013K-s004 [file CB-006-D5CB00013K-s004.pdf]

Supplementary Information (SI) for RSC Chemical Biology.
This journal is © The Royal Society of Chemistry 2025

Supplementary information

Exploring Sequence- and Structure-based Fitness Landscapes to Enhance Thermal Resistance and Activity of Endoglucanase II with Minimal Experimental Effort

Atul Kumar[1], Alexander-Maurice Illig[1], Nicolas de la Vega Guerra[1], Francisca C. Leiva[1], Mehdi D. Davari[2], Ulrich Schwaneberg[1,*]

[1]*Institute of Biotechnology, RWTH Aachen University, Worringerweg 3, Aachen, 52074, Germany*
[2]*Department of Bioorganic Chemistry, Leibniz Institute of Plant Biochemistry, Weinberg 3, Halle, 06120, Germany*

[*]*Corresponding author: u.schwaneberg@biotec.rwth-aachen.de*

Contents

1 **Improvements in thermostability of cellulases through protein engineering strategies** **3**

2 **Plasmid construction and mutagenesis of EGLII** **4**
2.1 Site-directed mutagenesis 5
2.1.1 Primers used for site-directed mutagenesis 5

3 **EVmutation: A Case Study on *Bacillus subtilis* Lipase A (BSLA)** **8**
3.1 Prediction of BSLA variants 8
3.2 Plasmid Construction and Mutagenesis 8
3.2.1 Primers used for site-directed mutagenesis 10
3.3 Cloning and expression of BSLA variants 10
3.4 Enzymatic activity of BSLA variants after thermal stress 11
3.5 Half-life of BSLA variants 11

4 **Correlation between predicted energy ($\Delta\Delta G$ and ΔE) with T_m and normalized activity** **13**

5 **Comparative analysis of wild-type EGLII and top-performing single- and double-substituted variant** **14**

6 **Oligomerization analysis of wild-type EGLII and top-performing single- and double-substituted variant** **15**
6.1 SDS PAGE analysis 15
6.2 Native-PAGE analysis 15
6.3 DLS measurements 16
6.3.1 DLS under native buffer conditions 17
6.3.2 DLS after thermal stress 17
6.3.3 DLS under high salt conditions 17

7 **SDS-PAGE analysis of predicted EGLII variants from FoldX and EVmutation (single and double substituted)** **19**

8 **Half-lives of predicted EGLII variants from FoldX and EVmutation (single and double substituted)** **20**

9 **Enzymatic activity of predicted EGLII variants without thermal stress** **22**

10 **Enzymatic activity of predicted EGLII variants after thermal stress at** 76 °C **23**

11 **Thermal unfolding curves of predicted EGLII variants from FoldX and EVmutation (single and double substituted)** **24**

12 **ConSurf analysis of predicted EGLII positions (FoldX and EVmutation)** **36**

13 **References** **37**

List of Tables

1 Overview of protein engineering approaches employed to improve the thermostability of cellulases 3
2 Sequences of primers or predicted EGLII variants, designed using Clone Manager 9. 5
3 Top 20 BSLA variants predicted by EVmutation. 8
4 Sequences of primers for predicted BSLA variants, designed using Clone Manager 9. 10
5 Estimated half-lives of BSLA variants at 50 °C. 12
6 Pearson and Spearman correlation coefficients between predicted energy values ($\Delta\Delta G$,ΔE_{single} and ΔE_{double}) and normalized activity after thermal stress and melting temperature (T_m). 13
7 Half-lives of the predicted EGLII variants by FoldX and EVmutation. 20
8 Conservation and exposure of predicted EGLII positions 36

List of Figures

1 Representation of plasmid construct of EGLII 4
2 Plasmid map of BSLA construct 9
3 Normalized activity of BSLA variants relative to wild type before (A) and after thermal stress (B) 11
4 Half-lives of the predicted BSLA variants 12
5 Correaltion of predictive energy values from FoldX and EVmutation with experimentally determined T_m (A) and normalized enzymatic activity after thermal stress at 75 °C (B) 13
6 Thermal performance comparison of wild-type EGLII with the top-performing single-substitution variant Q289G and double-substitution variant S180T/Q289G. 14
7 SDS-PAGE analysis of purified EGLII WT(WT), Q289G (1) and S180T/Q289G (2) under reducing and non-reducing conditions 15
8 Native PAGE analysis of EGLII wild type (WT), Q289G (1) and S180T/Q289G (2) 16
9 Dimensions of the crystallographic structure of EGLII monomer 16
10 Estimated hydrodynamic diameters of EGLII variants under native buffer conditions (Range 1) . . 17
11 Estimated hydrodynamic diameters of EGLII variants after one hour incubation at 75 °C (Range 1) 17
12 Estimated hydrodynamic diameters of EGLII variants in presence of high salt concentrations (Range 1) 18
13 SDS-PAGE analysis of predicted variants from FoldX and EVmutation (single- and double-substituted) 19
14 SDS-PAGE analysis of EGLII wild-type and variants T110N and N299S. 19
15 Half-lives of single-substituted EGLII variants predicted by FoldX. 20
16 Half-lives of single-substituted EGLII variants predicted by EVmutation 21
17 Half-lives of double-substituted EGLII variants predicted by EVmutation 21
18 Normalized enzymatic activity of FoldX-predicted single-substituted variants without thermal stress. 22
19 Normalized enzymatic activity of EVmutation-predicted single-substituted variants without thermal stress. 22
20 Normalized enzymatic activity of EVmutation-predicted double-substituted variants without thermal stress. 22
21 Normalized enzymatic activity of FoldX-predicted single-substituted variants after 1-hour incubation at 76 °C. 23
22 Normalized enzymatic activity of EVmutation-predicted single-substituted variants after 1-hour incubation at 76 °C. 23
23 Normalized enzymatic activity of EVmutation-predicted double-substituted variants after 1-hour incubation at 76 °C. 23
24 Thermal unfolding curves of FoldX single-substituted variants 24
25 Thermal unfolding curves of FoldX single-substituted variants 25
26 Thermal unfolding curves of FoldX single-substituted variants 26
27 Thermal unfolding curves of FoldX single-substituted variants 27
28 Thermal unfolding curves of EVmutation single-substituted variants 28
29 Thermal unfolding curves of EVmutation single-substituted variants 29
30 Thermal unfolding curves of EVmutation single-substituted variants 30
31 Thermal unfolding curves of EVmutation single-substituted variants 31
32 Thermal unfolding curves of EVmutation double-substituted variants 32
33 Thermal unfolding curves of EVmutation double-substituted variants 33
34 Thermal unfolding curves of EVmutation double-substituted variants 34
35 Thermal unfolding curves of EVmutation double-substituted variants 35
36 ConSurf analysis of EGLII 36

1. Improvements in thermostability of cellulases through protein engineering strategies

Table 1: Overview of protein engineering approaches employed to improve the thermostability of cellulases

Strategy	Enzyme	Improvement	Reference
Directed evolution	*Trichoderma reesei* endoglucanase III (Cel12A)	Improved pH stability (4.4-8.8), thermotolerance (fully active at 55 °C for 30 minutes), and 1.4-fold increase in catalytic efficiency	[1]
Directed evolution	*Clostridium thermocellum* Cel8A	S329G led to 7 °C increase in T_m and 8-fold improvement in $t_{1/2}$ at 85 °C	[2]
Directed evolution	*Chaetomium thermophilum* cellobiohydrolase II	Higher optimum temperature (60 °C), improved pH stability (up to pH 6), and maintained more than 50% activity after 1-hour incubation at 80 °C	[3]
Directed evolution	Chimeric fungal family 6 cellobiohydrolase (HJPlus)	Thermostable Cel6A variant with a half-life ($t_{1/2}$) of 280 minutes at 75 °C and T_{50} of 80.1 °C, a 15 °C increase over *Humicola insolens* Cel6A and a 20 °C increase over *Hypocrea jecorina* Cel6A	[4]
Directed evolution	β-glucosidase Ks5A7 from a Kusaya gravy metagenome	8640-fold improvement in half-life at 50 °C	[5]
Directed evolution	*Clostridium thermocellum* β-glucosidase	Increase in T_m by 6.4 °C	[6]
Rational design	*Melanocarpus albomyces* cellobiohydrolase Cel7B	Introduction of an additional disulphide bridge increased T_m up to 4 °C and improved activity towards microcrystalline cellulose at 75 °C	[7]
Rational design	*Thermotoga maritima* β-1,4-endoglucanase Cel12B	Rational design based on sequence alignment and homology modelling led to mutants maintaining 80 % and 90.5 % of initial activity after 8 hours at 80 °C	[8]
Rational design	*Chaetomium thermophilum* β-1,4-endoglucanase CTendo45	Improved residual activity over a range of 60 to 90 °C through structure-based rational design, involving mutation of conserved and noncatalytic residues, optimization of N-glycosylation sites, and combining both approaches	[9, 10, 11]
Rational design	*Penicillium verruculosum* endoglucanase II	Introduction of disulphide bridges led to almost 20 % increase in specific activity and improved residual activity at 80 °C	[12]
Rational design	*Penicillium verruculosum* endoglucanase II	Substitution of residues with proline improved $t_{1/2}$ by 2.4-fold at 80 °C	[13]
Rational design	*Penicillium verruculosum* Cel7A cellobiohydrolase	Substitution of residues with proline improved $t_{1/2}$ by 3.4 times at 60 °C	[14]
Combined strategy	Various fungal glycosyl hydrolase Cel7A genes	Biased clique shuffling created a library of chimeras with 86% activity and 51 showing improved thermostability	[15]
Combined strategy	Consensus mutation library from five thermophilic fungal cellobiohydrolase II (CBH2)	Improved thermostability of *Phanerochaete chrysosporium* CBH2, retaining activity at 50 °C for 72 hours	[16]
Combined strategy	*Hypocrea pseudokoningii* Cel7B endoglucanase	Enhanced T_m by 10 °C through comparison with thermostable homologues	[17]
Combined strategy	Chimeric enzyme from cellulases of *Geobacillus sp. 70PC53* GsCelA and *Bacillus* homolog BsCel5A	Created using SCHEMA, retains 40% activity at 90 °C	[18]
Combined strategy	*Hypocrea jecorina* Cel7A cellobiohydrolase	Increased T_m by 10.4 °C and 44-fold half-life increase using structural and dynamic approaches	[19]
Combined strategy	Chimeric enzymes from thermophilic GH5 endoglucanase Egl5A and mesophilic *Stegonsporium opalus* Cel5	Two hybrids showed significant increases in T_{opt} (10 and 20 °C), T_{50} (15 and 19 °C), T_m (16.5 and 22.9 °C), and $t_{1/2}$ (240- and 650-fold at 55 °C)	[20]
Combined strategy	*Penicillium verruculosum* endo-β-1,4-glucanase	KnowVolution strategy yields a variant with a 5.5-fold longer half-life at 75 °C and 7.7 °C higher T_m	[21]
Combined strategy	*Penicillium verruculosum* endo-β-1,4-glucanase	Constraint network analysis reduced screening effort to 40% with up to 2-fold improved thermostable variants	[22]

2. Plasmid construction and mutagenesis of EGLII

pBSYA1S1Z::EGLII : pBSYA1S1Z is a shuttle vector (pUC Ori and ARS) with zeocin resistance as a selection marker. EGLII represents the codon-optimized synthetic endo-β-Glucanase gene from *Penicillium verruculosum* (UniProtKB: A0A1U7Q1U3) cloned between *XhoI* and *NotI* restriction sites. The gene is expressed with the GAP promoter (P_{GAP}) and secreted into the supernatant by the α-signal peptide (alpha SP).

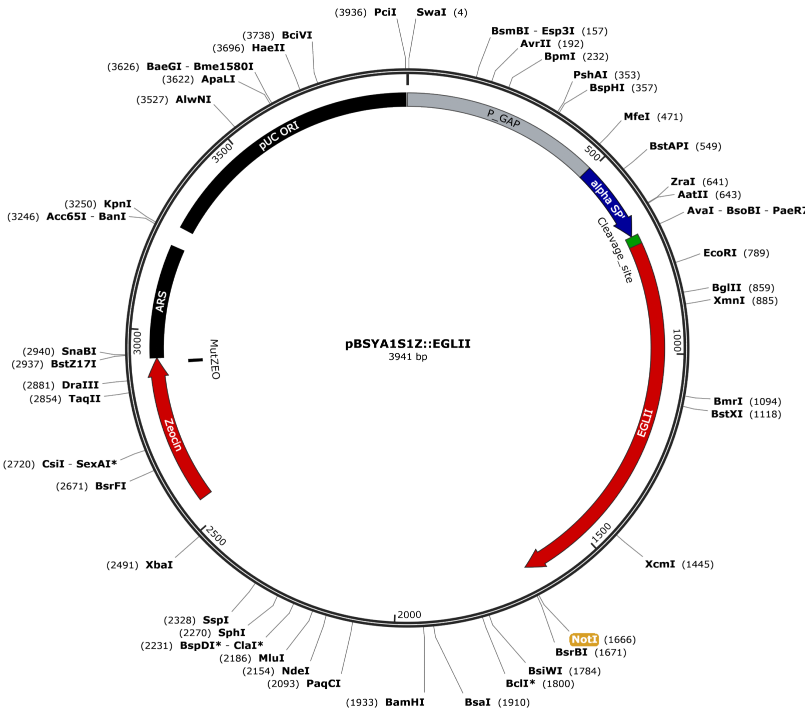

Figure 1: Representation of plasmid construct of EGLII

The EGLII gene was codon optimized for *Pichia pastoris*.

CTCGAGAAGAGAGAGGCCGAAGCTGCTAACTCTAAAGAGGTTAAGAAGAGAGCTTCCTCCTTCGA
ATGGTTCGGTTCTAACGAATCTGGTGCTGAATTCGGTTCCGGTAACATTCCAGGTGTTGAGGGTAC
TGACTACACTTTCCCAAACACTACTGCTATCCAGATCTTGATCGACGCTGGTATGAACATCTTCAGA
GTTCCATTCTTGATGGAAAGAATGATCCCAACTGAGATGACTGGTTCCTTGGACACTGCTTACTTCG
AAGGTTACTCCGAGGTTTTGAACTACATCACTGGTAAGGGTGCTCACGCTGTTGTTGATCCACACA
ACTTCGGTAGATATTACGGTACTCCAATCTCCTCCACTTCCGACTTCCAAACTTTCTGGTCTACTTTG
GCTTCCCAGTTCAAGTCCAACGACTTGGTTATCTTCGACACTAACAACGAGTACCACGACATGGAC
GAGTCCGTTGTTGTTGCTTTGAACCAGGCTGCTATCGACGGTATTAGAGATGCTGGTGCTACTACTC
AGTACATCTTCGTTGAAGGTAACGCTTACTCCGGTGCTTGGACTTGGACTACTTACAACACTGCTAT
GGTTAACTTGACTGACCCATCCGACTTGATCGTTTACGAGATGCACCAATACTTGGACTCCGACGG
TTCTGGTACTTCCGACCAATGTGTTTCTTCCACTGTTGGTCAAGAGAGAGTTGTTGACGCTACTACT
TGGTTGCAGTCCAACGGAAAGTTGGGTATCTTGGGTGAATTTGCTGGTGGTGCTAACTCCGTTTGTG
AAGAGGCTGTTGAGGGAATGTTGGATTACTTGGCTGAGAACTCCGACGTTTGGTTGGGTGCTTCTT
GGTGGTCTGCTGGTCCATGGTGGCAAGATTACATCTACTCTATGGAACCACCAAACGGTATCGCTT
ACGAGTCCTACTTGTCCATCTTGGAGACTTACTTCTAAGCGGCCGC

2.1. *Site-directed mutagenesis*

EGLII variants predicted by EVmutation and FoldX were generated by site-directed mutagenesis (SDM) using a two-step polymerase chain reaction (PCR) method [23]. The following components were used for the SDM PCR: PCRBIO VeriFi™ Polymerase mix (containing VeriFi™ Polymerase, dNTPs, and enhancers at 1X concentration), primers (0.4 µM), template DNA (20 ng), and a thermal cycler. The PCR conditions were as follows: First step: 98 °C for 60 s (1 cycle); 98 °C for 15 s, 60 °C for 15 s and 72 °C for 150 s (4 cycles). Second step: 98 °C for 60 s (1 cycle); 98 °C for 15 s, 60 °C for 15 s and 72 °C for 150 s (25 cycles); 72 °C for 10 min (1 cycle).

After the PCR, DpnI (20 U; New England Biolabs) was added for the digestion of template DNA, and the mixture was incubated overnight at 37 °C. The DpnI digested PCR products were purified using a NucleoSpin® Extract II Purification Kit (Macherey-Nagel, Germany), transformed into *E. coli* DH5α, and plated on low-salt LB agar plates (containing tryptone $10\,g\,L^{-1}$, NaCl $5\,g\,L^{-1}$, yeast extract $5\,g\,L^{-1}$ and agar $15\,g\,L^{-1}$) with $50\,\mu g\,mL^{-1}$ zeocin. The generated recombinant variants were confirmed by sequencing at Eurofins MWG (Ebersberg, Germany). The plasmids containing the mutations were then transformed into *P. pastoris* BG11 and plated on YPD agar plates (containing peptone $20\,g\,L^{-1}$, D-glucose $20\,g\,L^{-1}$, yeast extract $10\,g\,L^{-1}$ and agar $15\,g\,L^{-1}$) with $100\,\mu g\,mL^{-1}$ zeocin.

2.1.1. *Primers used for site-directed mutagenesis*

Table 2: Sequences of primers or predicted EGLII variants, designed using Clone Manager 9.

S. No.	Name	Sequence (5' to 3')
1	D138N_F	GGTTATCTT**CAA**CACTAACAACGAGTACC
2	D138N_R	GGTACTCGTT**GTT**AGTGTTGAAGATAACC
3	E205M_F	GATCGTTTAC**ATG**ATGCACCAATAC
4	E205M_R	TTGGTGCAT**CAT**GTAAACGATCAAG
5	D138C_F	GGTTATCTTC**TGC**ACTAACAACGAG
6	D138C_R	CGTTGTTAGT**GCA**GAAGATAACCAAG
7	E20M_F	TCGGTTCTAAC**ATG**TCTGGTGCTG
8	E20M_R	CAGCACCAGA**CAT**GTTAGAACCG
9	D138V_F	GGTTATCTTC**GTG**ACTAACAACGAG
10	D138V_R	CTCGTTGTTAGT**CAC**GAAGATAACC
11	D138L_F	GGTTATCTTC**CTG**ACTAACAACGAGTACC
12	D138L_R	CTCGTTGTTAGT**CAG**GAAGATAACC
13	E205C_F	ATCGTTTAC**TGT**ATGCACCAATACTTGGAC
14	E205C_R	TTGGTGCAT**ACA**GTAAACGATCAAG
15	E205Q_F	TGATCGTTTAC**CAG**ATGCACCAATAC
16	E205Q_R	AGTATTGGTGCAT**CTG**GTAAACG
17	E205L_F	GATCGTTTAC**TTG**ATGCACCAATAC
18	E205L_R	TTGGTGCAT**CAA**GTAAACGATCAAG
19	S280M_F	GTTGGGTGCT**ATG**TGGTGGTCTG
20	S280M_R	AGACCACCA**CAT**AGCACCCAACC
21	S280L_F	GTTGGGTGCT**TTG**TGGTGGTCTG
22	S280L_R	GACCACCA**CAA**AGCACCCAACC
23	D138S_F	GGTTATCTTC**AGC**ACTAACAACGAG
24	D138S_R	CTCGTTGTTAGT**GGA**GAAGATAACC
25	D138A_F	TGGTTATCTTC**GCC**ACTAACAACG
26	D138A_R	CTCGTTGTTAGT**GGC**GAAGATAACC
27	E20L_F	TCGGTTCTAAC**TTG**TCTGGTGCTG
28	E20L_R	TCAGCACCAGA**CAA**GTTAGAACCG
29	S114D_F	CCAATCTCC**GAC**ACTTCCGACTTC

30	S114D_R	AGTCGGAAGT**GTC**GGAGATTGG
31	E175M_F	CATCTTCGTT**ATG**GGTAACGCTTACTC
32	E175M_R	TAAGCGTTACC**CAT**AACGAAGATGTACTG
33	G181R_F	CGCTTACTCC**AGA**GCTTGGACTTG
34	G181R_R	TCCAAGTCCAAGC**TCT**GGAGTAAG
35	D138T_F	GGTTATCTTC**ACT**ACTAACAACGAGTACC
36	D138T_R	GGTACTCGTTGTTAGT**AGT**GAAGATAACC
37	E205A_F	GATCGTTTAC**GCT**ATGCACCAATAC
38	E205A_R	ATTGGTGCAT**AGC**GTAAACGATCAAG
39	E20I_F	TCGGTTCTAAC**ATC**TCTGGTGCTG
40	E20I_R	CAGCACCAGA**GAT**GTTAGAACCG
41	P111I_F	TACGGTACT**ATC**ATCTCCTCCAC
42	P111I_R	GTGGAGGAGAT**GAT**AGTACCG
43	T236A_F	TTGACGCTACT**GCT**TGGTTGC
44	T236A_R	CAACCA**AGC**AGTAGCGTCAAC
45	Q289A_F	CCATGGTGG**GCT**GATTACATCTAC
46	Q289A_R	ATGTAATC**AGC**CCACCATGGAC
47	P111V_F	TTACGGTACT**GTT**ATCTCCTCCAC
48	P111V_R	GAAGTGGAGGAGAT**AAC**AGTACCG
49	T110N_F	GATATTACGGT**AAC**CCAATCTCCTCC
50	T110N_R	AGGAGATTGG**GTT**ACCGTAATATCTACCG
51	E81A_F	CTGCTTACTTC**GCA**GGTTACTC
52	E81A_R	CGGAGTAACC**TGC**GAAGTAAG
53	D164G_F	GTATTAGA**GGT**GCTGGTGCTAC
54	D164G_R	AGCACCAGC**ACC**TCTAATACC
55	V150L_F	GACGAGTCC**TTG**GTTGTTGCTTTG
56	V150L_R	CAAAGCAACAAC**CAA**GGACTCG
57	Y188V_F	GGACTACT**GTC**AACACTGCTATG
58	Y188V_R	CATAGCAGTGTT**GAC**AGTAGTCC
59	Q239K_F	CTTGGTTG**AAG**TCCAACGGAAAG
60	Q239K_R	TCCGTTGGA**CTT**CAACCAAGTAG
61	L201K_F	CCCATCCGAC**AAG**ATCGTTTAC
62	L201K_R	CGTAAACGAT**CTT**GTCGGATGG
63	S123T_F	CTTTCTGG**ACT**ACTTTGGCTTC
64	S123T_R	GAAGCCAAAG**TAG**TCCAGAAAG
65	A153D_F	CCGTTGTTGTT**GAC**TTGAACCAG
66	A153D_R	CCTGGTTCAA**GTC**AACAACAACG
67	P111I_F	TACGGTACT**ATC**ATCTCCTCCAC
68	P111I_R	AGTGGAGGAGAT**GAT**AGTACCG
69	D164A_F	GTATTAGA**GCT**GCTGGTGCTAC
70	D164A_R	AGCACCAGC**AGC**TCTAATACC
71	A178S_F	GGTAAC**TCC**TACTCCGGTGCTTG
72	A178S_R	ACCGGAGTA**GGA**GTTACCTTC
73	S180T_F	AACGCTTAC**ACT**GGTGCTTGG
74	S180T_R	AAGCACC**AGT**GTAAGCGTTACC
75	V226I_F	CTTCCACT**ATC**GGTCAAGAGAG
76	V226I_R	CTCTTGACC**GAT**AGTGGAAGAAAC

77	T236Q_F	GACGCTACT**CAA**TGGTTGCAGTC
78	T236Q_R	CTGCAACCA**TTG**AGTAGCGTCAAC
79	N299S_F	AACCACCA**TCT**GGTATCGCTTACG
80	N299S_R	AGCGATACC**AGA**TGGTGGTTCC

3. EVmutation: A Case Study on *Bacillus subtilis* Lipase A (BSLA)

To evaluate the broader applicability of EVmutation, the method was applied to *Bacillus subtilis* Lipase A, a mesophilic serine hydrolase (T_m - 49.5 °C). The top 20 variants ranked by EVmutation (based on ΔE) were selected for experimental validation and these variants were assessed for thermal resistance by subjecting to thermal stress at 50 °C for 30 minutes.

3.1. Prediction of BSLA variants

A multiple sequence alignment (MSA) was generated by performing a jackmmer search (HMMER 3.3.2)[24] against the UniProt Reference Clusters (UniRef) database UniRef100 [25, 26]. The search was initialized with the BSLA sequence using a bit score of half the sequence length. The MSA was then post-processed following the procedure outlined by Hopf et al.[27], removing sequences with more than 50 % gaps and positions with gaps in over 30 % of the sequences. Local biases and coupling terms were inferred using plmc (release 16 May 2018, available at https://github.com/debbiemarkslab/plmc) with regularization parameters lh = 0.01 and le = 0.2 (N 1), where N = 170 is the number of effective sites in the alignment. ΔE was then calculated for single-substituted variants. A table of the top 20 ranked single variants is given below and from the BSLA predicted variants, we can also observe that the variants are quite diverse similar to EGLII.

Table 3: Top 20 BSLA variants predicted by EVmutation.

Rank	Variant	ΔE
1	T47N	1.78
2	M137A	1.64
3	M137S	1.57
4	G111S	1.45
5	F17Y	1.32
6	Y139S	1.30
7	R147K	1.28
8	Y161S	1.21
9	M137T	1.19
10	G111N	1.15
11	S162N	0.94
12	H152S	0.93
13	W42L	0.92
14	T109V	0.89
15	M134E	0.88
16	R147N	0.79
17	M134Q	0.78
18	V149I	0.75
19	M137L	0.74
20	W42S	0.73

3.2. Plasmid Construction and Mutagenesis

The codon-optimized synthetic BSLA gene (UniProtKB: P37957) was cloned into the pET-22b(+) vector using *NcoI* and *XhoI* restriction sites (see codon optimized sequence of BSLA). The gene was expressed under the control of the T7 promoter and targeted to the periplasm using the signal peptide encoded by the vector. This plasmid (pET-22b(+)-bsla-his) (Figure 2) served as the template for site-directed mutagenesis (SDM), performed using the PCRBIO VeriFi™ polymerase mix. The following components were used for the SDM PCR: PCRBIO VeriFi™ Polymerase mix (containing VeriFi™ Polymerase, dNTPs, and enhancers at 1X concentration), primers (0.4 µM), template DNA (20 ng), and a thermal cycler. The PCR conditions were as follows:First step:98 °C for 30 s (1 cycle); 98 °C for 10 s, 50 °C for 30 s, and 72 °C for 180 s (2 cycles). Second step:98 °C for 30 s (1 cycle); 98 °C for 10 s, 50 °C for 30 s, and 72 °C for 180 s (25 cycles); 72 °C for 10 min (1 cycle). Primers for each substitution is listed in Table 4.

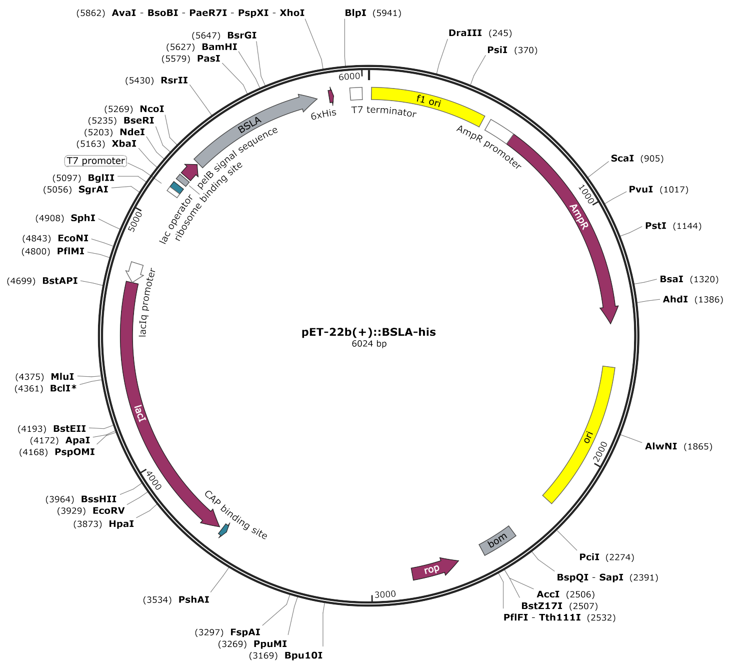

Figure 2: Plasmid map of BSLA construct

The BSLA gene was codon optimized for *Escherichia coli* .
CCATGGGCGCTGAACATAATCCCGTCGTAATGGTTCACGGGATCGGCGGCGCTTCGTTCAACTTTG
CAGGTATCAAGTCCTATTTGGTCAGCCAAGGTTGGAGCCGTGATAAACTGTACGCAGTTGACTTCT
GGGATAAGACCGGCACGAACTATAACAACGGTCCGGTTCTGAGCCGTTTTGTTCAGAAGGTGCTGG
ACGAGACTGGTGCTAAAAAGGTGGACATCGTGGCGCACAGCATGGGTGGCGCGAACACCTTGTAC
TATATCAAGAACCTGGACGGCGGTAATAAAGTGGCCAATGTTGTCACCCTGGGTGGCGCGAACCG
CCTTACCACCGGCAAGGCGCTGCCGGGTACGGATCCGAATCAGAAAATCCTGTACACCAGCATTTA
TTCCTCTGCCGATATGATTGTGATGAACTACCTCTCTCGCCTGGACGGTGCGCGTAATGTGCAGAT
TCATGGTGTTGGTCACATCGGCTTGCTGTACTCCAGCCAAGTTAATAGCCTGATTAAAGAAGGTTT
AAATGGCGGTGGCCAAAACACCAACGAGAACCTGTACTTCCAATCCCACCACCATCATCATCATTA
ACTCGAG

3.2.1. *Primers used for site-directed mutagenesis*

Table 4: Sequences of primers for predicted BSLA variants, designed using Clone Manager 9.

Primer Name	Sequence
F17Y_F	GGCGCTTCG**TAC**AACTTTGCAG
F17Y_R	CTGCAAAGTT**GTAC**GAAGCGCC
W42S_F	GTTGACTTC**CAG**CGATAAGACCG
W42S_R	CGGTCTTAT**CGC**TGAAGTCAAC
W42L_F	GTTGACTTC**CTG**GATAAGACCG
W42L_R	CGGTCTTAT**CCAG**GAAGTCAAC
T47N_F	AAGACCGGC**AAC**AACTATAAC
T47N_R	GTTATAGT**TGTTG**CCGGTCTT
T109V_F	AACCGCCT**TGT**GACCGGCAAG
T109V_R	CTTGCCGGT**CACA**AGGCGGTT
G111S_F	CTTACCACC**AGC**AAGGCGCTG
G111S_R	CAGCGCCTT**GCT**GGTGGTAAG
G111N_F	CTTACCACC**AAC**AAGGCGCTG
G111N_R	CAGCGCCTT**GTT**GGTGGTAAG
M134E_F	CCTCTGCCGAT**GAA**ATTGTGATG
M134E_R	CATCACAAT**TTT**CATCGGCAGAGG
M134Q_F	CTCTGCCGAT**CAG**ATTGTGATG
M134Q_R	CATCACAAT**CTG**ATCGGCAGAG
M137A_F	ATGATTGTG**GCG**AACTACCTC
M137A_R	GAGGTAGTT**CGC**CACAATCAT
M137S_F	ATGATTGTG**AGC**AACTACCTC
M137S_R	GAGGTAGTT**GCT**CACAATCAT
M137T_F	ATGATTGTG**ACC**AACTACCTC
M137T_R	GAGGTAGTT**GGT**CACAATCAT
M137L_F	ATGATTGTG**CTG**AACTACCTC
M137L_R	GAGGTAGTT**CAG**CACAATCAT
Y139S_F	GTGATGA**ACAGC**CTCTCTCGC
Y139S_R	GCGAGAGAG**GCT**GTTCATCAC
R147K_F	GACGGTGCG**AAAAA**TGTGCAG
R147K_R	CTGCACAT**TTTTC**GCACCGTC
R147N_F	GACGGTGCG**AACA**ATGTGCAG
R147N_R	CTGCACAT**TGTT**CGCACCGTC
V149I_F	GCGCGTAAT**ATT**CAGATTCATGG
V149I_R	CCATGAATCT**GAAT**ATTACGCGC
H152S_F	GTGCAGATT**TCC**GGTGTTGGTC
H152S_R	GACCAACAC**CGG**AAATCTGCAC
Y161S_F	GGCTTGCT**GAGC**TCCAGCCAA
Y161S_R	TTGGCTGGA**GCTC**AGCAAGCC
S162N_F	CTTGCTGT**ACA**ACAGCCAAGTT
S162N_R	AACTTGGC**TGTT**GTACAGCAAG

3.3. *Cloning and expression of BSLA variants*

Escherichia coli DH5α (Agilent Technologies, Santa Clara, USA) was used as the cloning host, and *Escherichia coli* BL21 DE3 Gold (Agilent Technologies, Santa Clara, USA) was used as the expression host. BSLA variants were cultured in 96-well F-bottom polystyrene microtiter plates (MTPs) (Greiner, Frickenhausen, Germany) and and expressed in 96-well V-bottom polystyrene microtiter plates (MTPs) (Corning Inc., 2 Alfred Rd, Kennebunk, ME 04043, United States) using an MTP shaker (Infors HT Multitron, Bottmingen, Switzerland). Luria-Bertani (LB) media (0.5 % w/v yeast extract, 1 % w/v tryptone, and 1 % w/v NaCl) supplemented with 100 $\mu g\,mL^{-1}$ ampicillin was used for cell culture. Modified Auto-induction media (2.4 % w/v yeast extract, 1.2 % w/v tryptone, and 0.5 % w/v glycerol, 0.05 % w/v glucose, 0.2 % w/v lactose, 9 % v/v KPi Buffer (pH 7, 1M)) supplemented with 100 $\mu g\,mL^{-1}$ ampicillin was used for protein expression. Colonies picked from LB agar plates were used to inoculate preculture (150 µL per well, 37 °C, 900 rpm, 16 hours, and 70 % humidity). The main ex-

pression culture (150 µL per well, 30 °C, 900 rpm, 16 hours, and 70 % humidity) was inoculated with a volume of 5 µL from the preculture. The cell pellet was separated from the cell culture by centrifugation (Eppendorf 5810R; 4 °C, 3220 x g, 20 minutes), and 150 µL of lysis buffer (0.02 % v/v DNAase, 1 % v/v $MgCl_2$ (0.5 M), and 0.05 % w/v lysozyme in 50 mM Triethanolamine Buffer (TEA buffer, pH 7.4)) was added per well. The MTP containing cell pellet and lysis buffer was incubated (37 °C,1 hour, 900 rpm, and 70 % humidity) using an MTP shaker (Infors HT Multitron, Bottmingen, Switzerland). The cell debris was separated by centrifugation (Eppendorf 5810R; 4 °C, 3220 x g, 20 minutes) and lipase containing supernatant was used for further analysis.

3.4. Enzymatic activity of BSLA variants after thermal stress

p-Nitrophenyl butyrate (pNPB) assay in MTP format was used to assess the hydrolytic activity of BSLA [28]. The assay is based on the hydrolysis of p-Nitrophenyl butyrate into p-nitrophenolate which is detected at 410nm. The lipase containing supernatant was diluted 10-fold in TEA buffer (50 mM) and then incubated at high temperature (50 °C) without the substrate. To evaluate the activity of BSLA variants after thermal stress , the temperature of 50 °C for 30 minutes was chosen as it reduced the wild type enzyme activity to approximately 30 %. Enzymatic activity was measured before (T_0) and after (T_{30}) thermal stress by adding 100 µL of freshly prepared substrate solution (TEA buffer containing 0.5 mM pNPB and 10 % v/v acetonitrile) to each well and monitoring the release of p-nitrophenolate at 410 nm using CLARIOstar Plus microplate reader from BMG Labtech (Ortenberg, Germany). Activity was quantified at room temperature by the slope of absorbance over 4 minutes (13 kinetic cycles, 5 flashes/well). Each variant was analyzed in at least three biological replicates.

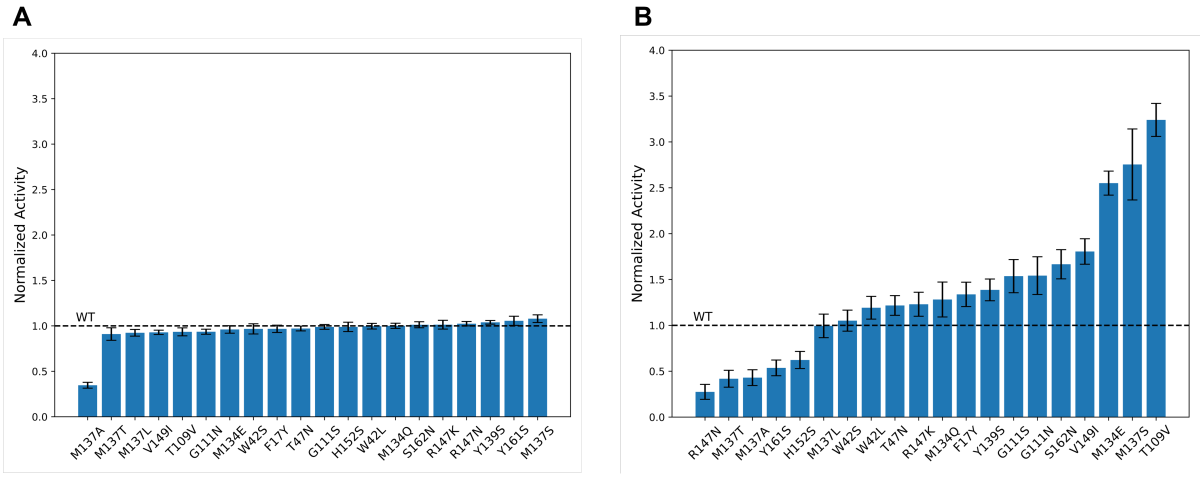

Figure 3: Normalized activity of BSLA variants relative to wild type before (A) and after thermal stress (B)

The results showed that, under non-stress conditions, all variants except M137A exhibited similar activity to the WT (Figure 3A). After thermal stress, 13 out of 20 variants displayed increased residual activity, yielding a hit rate of 65 % (Figure 3B). Notably, three variants exhibited more than 2.5-fold improvement, with T109V showing the highest (3.2-fold). These results demonstrate that EVmutation performs comparably—or even better—for the enzyme with lower or moderate thermostability, underscoring the method's generalizability to other enzyme classes.

3.5. Half-life of BSLA variants

The half-lives were also determined for the predicted 20 variants. The diluted supernatant was incubated at 50 °C without the substrate for different time intervals ranging from 0 to 60 minutes. Subsequently, pNPB assay was used to assess the hydrolytic activity of incubated and non-incubated variants as described above. Residual activity was plotted against time, and half-lives were calculated via linear interpolation (Figure 4). All the measurements were conducted with atleast three biological replicates. Of the 20 tested variants, 16 showed improved half-lives compared to WT, with 7 variants exhibiting more than a two-fold increase in stability. The most thermostable variant, T109V, demonstrated a 5.4-fold improvement in half-life over WT (Table 5, Figure 4).

These findings collectively demonstrate that EVmutation can effectively enrich thermally resistant and active variants across different enzyme classes, provided sufficient sequences are available. The success observed with BSLA further supports the method's potential for widespread application in protein engineering workflows.

Table 5: Estimated half-lives of BSLA variants at 50 °C.

Variant	Half-Life (minutes)
R147N	3.19
H152S	6.88
M137T	7.25
Y161S	8.03
**WT**	**12.63**
M137L	13.19
R147K	15.48
W42S	16.02
W42L	16.56
Y139S	16.93
T47N	18.65
M134Q	19.73
F17Y	21.95
G111S	25.22
S162N	26.53
G111N	28.18
V149I	31.96
M134E	36.88
M137S	38.01
T109V	68.09

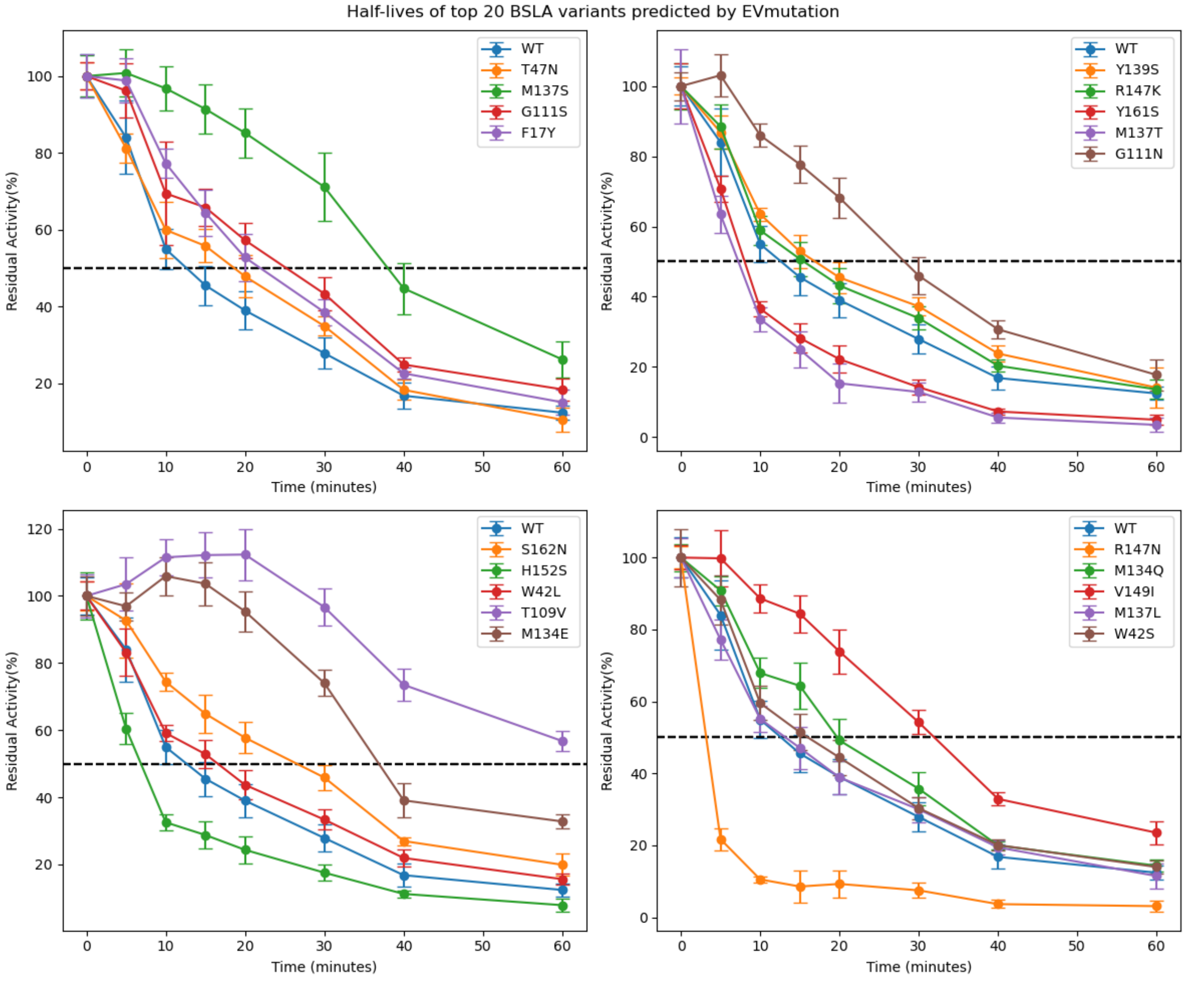

Figure 4: Half-lives of the predicted BSLA variants

4. Correlation between predicted energy ($\Delta\Delta G$ and ΔE) with T_m and normalized activity

To asess the predictive performance of FoldX and EVmutation in identifying functionally improved variants, we analyzed the relationship between computational/predictive energy scores——folding free energy differences ($\Delta\Delta G$) from FoldX and statistical energy differences (ΔE) from EVmutation—and two key experimental parameters: melting temperature (T_m) and enzymatic activity normalized to wild-type EGLII after thermal stress at 75 °C.

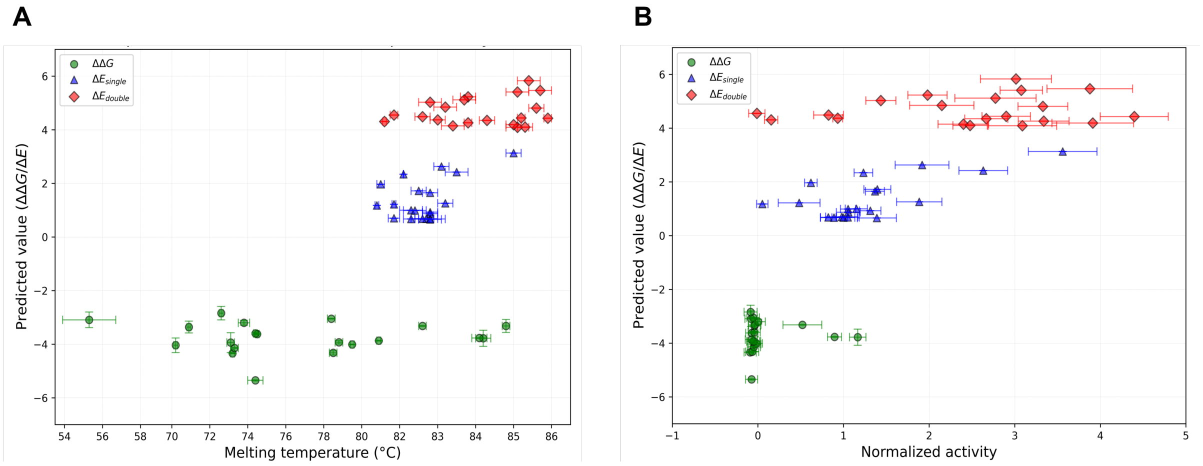

Figure 5: Correaltion of predictive energy values from FoldX and EVmutation with experimentally determined T_m (A) and normalized enzymatic activity after thermal stress at 75 °C (B)

Scatter plots were generated to visualize the distribution of predicted energy values against experimental measurements, and both Pearson and Spearman correlation coefficients were computed to quantify linear and rank-based associations, respectively. For single-substituted variants, the ΔE values from EVmutation exhibited moderate correlations with normalized activity (Pearson: 0.67; Spearman: 0.49) and T_m (Pearson: 0.41; Spearman: 0.19), outperforming FoldX ($\Delta\Delta G$), which showed weak or negligible correlations with either metric (see Table). In contrast, for double-substituted variants, EVmutation correlations were substantially weaker (see Table, ΔE_{double}). Although ΔE outperforms $\Delta\Delta G$ in terms of numerical correlation with experimental data, the differences in correlation strength are not statistically significant. This suggests that, on a purely numerical basis, ΔE is not substantially more predictive than $\Delta\Delta G$ when assessed using standard correlation metrics. Therefore, no statistically meaningful correlation could be established between the predicted energy scores and the experimental measurements based solely on Pearson or Spearman coefficients.

Table 6: Pearson and Spearman correlation coefficients between predicted energy values ($\Delta\Delta G$,ΔE_{single} and ΔE_{double}) and normalized activity after thermal stress and melting temperature (T_m).

	Normalized Activity		T_m	
**Predictor**	**Pearson**	**Spearman**	**Pearson**	**Spearman**
FoldX ($\Delta\Delta G$)	0.056941	0.123824	-0.115848	-0.006772
EVmutation (ΔE_{single})	0.674166	0.486080	0.405811	0.186272
EVmutation (ΔE_{double})	0.137420	-0.022556	0.202472	0.118134

However, a qualitative inspection of the scatter plots reveals a consistent pattern: variants predicted by EVmutation tend to cluster in regions associated with higher thermal stability and enhanced activity. In contrast, FoldX predictions are predominantly concentrated in areas of lower activity and moderate stability. These observations suggest that although ΔE may not dramatically surpass $\Delta\Delta G$ in quantitative correlation values, EVmutation is more effective in enriching for beneficial variants. This finding aligns with our experimental results and supports the practical utility of EVmutation as a tool for guiding protein engineering.

5. Comparative analysis of wild-type EGLII and top-performing single- and double-substituted variant

The EVmutation-predicted variants, Q289G and S180T/Q289G outperformed wild-type EGLII, retaining 3.6- and 4.4-fold higher activity after incubation at 75 °C. Their half-lives (104 ± 7 and 121 ± 15 minutes) and melting temperatures (85 °C and 85.9 °C) exceeded wild type (40 ± 2 minutes, 82.1 °C), with S180T/Q289G showing the highest improvement.

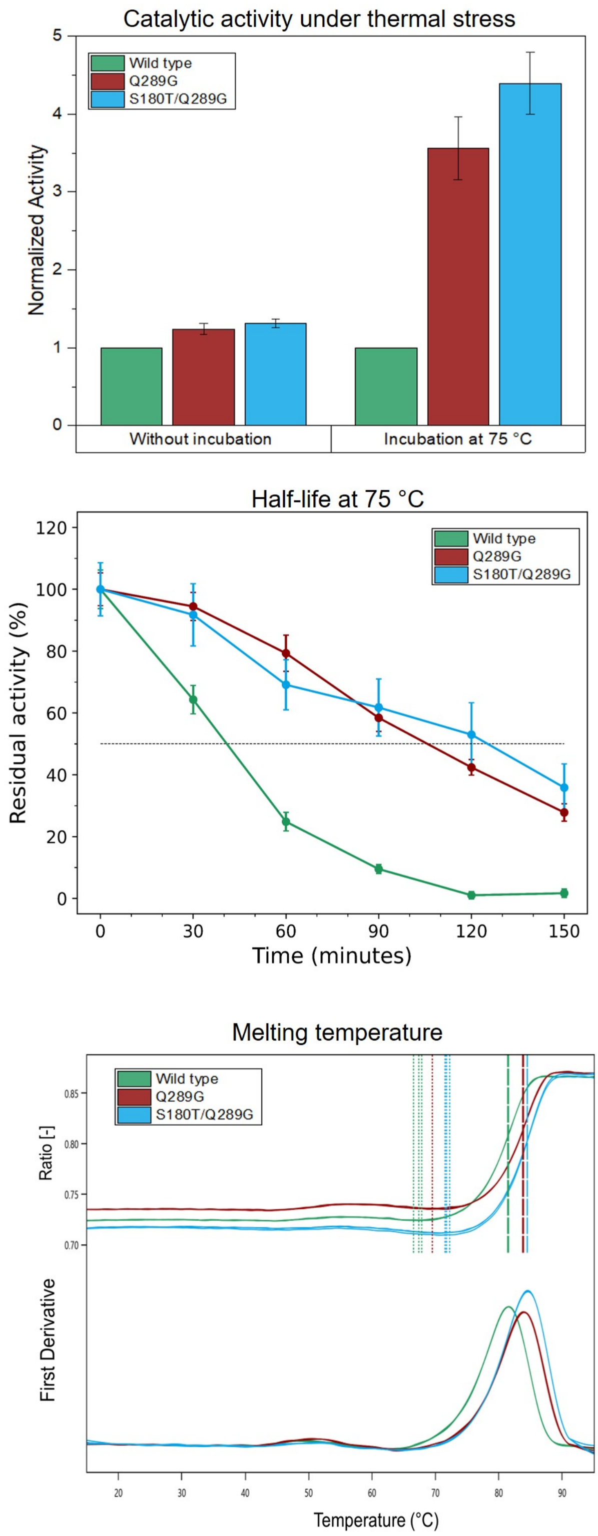

Figure 6: Thermal performance comparison of wild-type EGLII with the top-performing single-substitution variant Q289G and double-substitution variant S180T/Q289G.

6. Oligomerization analysis of wild-type EGLII and top-performing single- and double-substituted variant

To evaluate the oligomeric state and potential aggregation of the enzyme, the wild-type EGLII, along with the two best-performing variants—Q289G and the double mutant S180T/Q289G—were purified using anion exchange chromatography. For protein expression, *Pichia pastoris* cultures were grown in 100 mL YPD medium supplemented with 100 µg mL^{-1} Zeocin. The EGLII wild-type and variants were cultured for 96 hours in Erlenmeyer flasks (25 °C and 180 rpm). Following cultivation, the culture supernatant was clarified by centrifugation at 10,000×g for 30 minutes at 4 °C (Sorvall RC 6 Plus Centrifuge, Thermo Scientific). The resulting supernatant was concentrated to a final volume of 5 mL using a centrifugal filter unit (10kDa MWCO), and the culture medium was exchanged with 20 mM Tris-Cl buffer (pH 6.5; buffer A).

Protein purification was performed using fast protein liquid chromatography (FPLC) on an ÄKTAprime Plus system (GE Healthcare). The concentrated sample was loaded onto a 5 mL HiTrap DEAE FF anion exchange column pre-equilibrated with buffer A. The protein was eluted using a linear gradient of 15 % to 50 % Buffer B (20 mM Tris-Cl, pH 6.5, containing 1 M NaCl). Fractions corresponding to the expected size of 35 kDa were pooled and analyzed by SDS-PAGE under both reducing and non-reducing conditions (Figure**??**).Protein concentration of the purified enzyme variants was normalized by measuring the total protein concentration utilizing A280nm (NanoDrop™ 1000 spectrophotometer by Thermo Scientific™, Bremen, Germany), using the theoretical extinction coefficients determined based on the amino acid composition with ProtParam on the ExPASy server (81945 1 M^{-1} cm).

6.1. SDS PAGE analysis

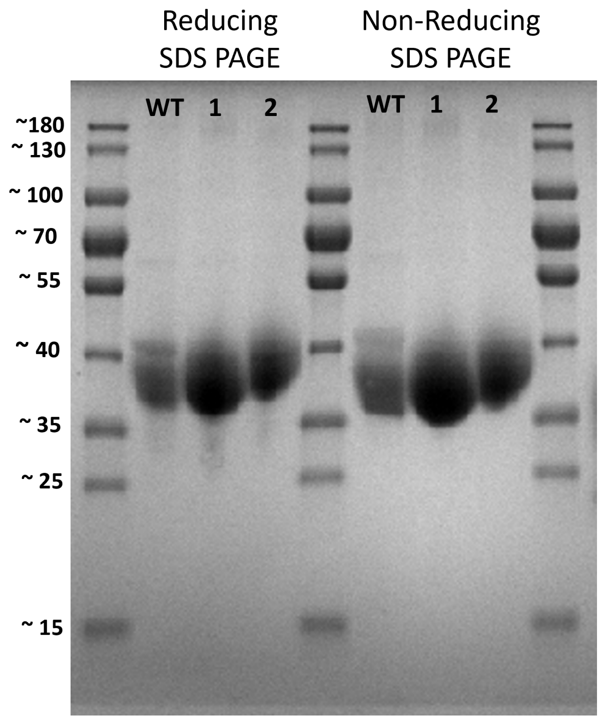

Figure 7: SDS-PAGE analysis of purified EGLII WT(WT), Q289G (1) and S180T/Q289G (2) under reducing and non-reducing conditions

SDS-PAGE analysis revealed that all three variants migrated as single bands at approximately 35 kDa under both reducing and non-reducing conditions. This indicates that the enzyme variants lack intermolecular disulfide-linked oligomers and maybe predominantly monomeric in nature.

6.2. Native-PAGE analysis

To further confirm the oligomeric state of EGLII and its variants, native PAGE analysis was performed. Although the bands appeared faint, we could observe prominent single bands for all variants in the native gel, further suggesting that EGLII and its variants exist predominantly in a monomeric state under native conditions.

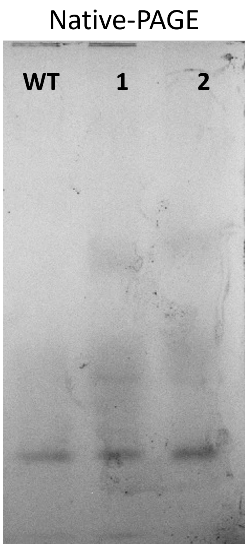

Figure 8: Native PAGE analysis of EGLII wild type (WT), Q289G (1) and S180T/Q289G (2)

6.3. DLS measurements

Dynamic light scattering (DLS) measurements were performed to assess the potential oligomerization and aggregation by assessing the hydrodynamic size and polydispersity of EGLII wild-type and its two best-performing variants (Q289G and S180T/Q289G). Measurements were conducted using the Wyatt DynaPro NanoStar, with data acquisition and analysis carried out using DYNAMICS 7 software. All samples were prepared at a concentration of 0.5 mg mL^{-1} in 20 mM Tris-Cl buffer (pH 6.5). Prior to measurement, each sample was filtered through 0.22 µM filters and equilibrated at 20 °C before measurement (except when assessing high temperature). DLS measurements were acquired using auto-attenuation mode (10 × 10-second acquisitions) and analyzed via cumulants and regularization (CONTIN) methods. Primary interpretation was based on the regularization method, which enables higher-resolution particle size distribution analysis. Particle sizes were evaluated in three dynamic ranges: Range 1 (0.2 nm to 20 nm), Range 2 (20 nm to 200 nm), and Range 3 (200 nm to 2000 nm). Size and dispersity assessments were based regularization algorithm with % number distribution weighting, which reflects the true population of particles in solution. Each sample was analyzed in atleast 8 replicates to ensure statistical reliability and reproducibility. Particles were considered monomeric if more than 95% of the number distribution fell within Range 1, consistent with the crystallographic dimensions of the EGLII monomer (5.5 × 4.7 × 3.7 nm; Figure 9). Minor peaks at higher diameters with negligible % number but higher intensity were attributed to trace aggregates or scattering artifacts, given the greater light-scattering power of larger particles.

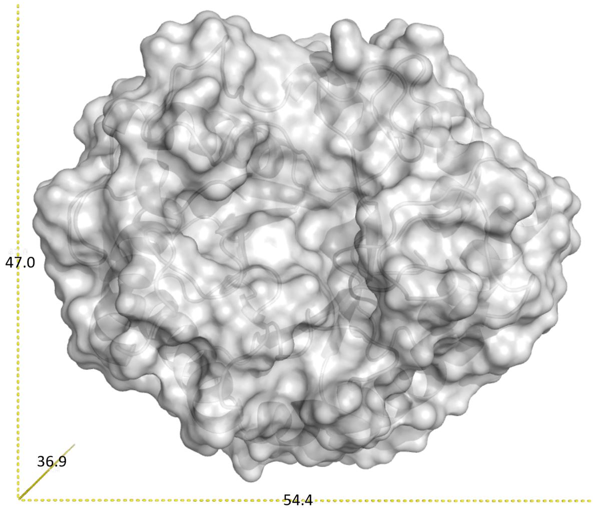

Figure 9: Dimensions of the crystallographic structure of EGLII monomer

6.3.1. *DLS under native buffer conditions*

DLS measurements revealed that all three enzyme variants—WT, Q289G, and S180T/Q289G—existed predominantly as monomeric species under native buffer conditions. More than 99% of particles by number were detected within the smallest size range (Range 1), confirming a monodisperse population. The average hydrodynamic diameters of the WT, Q289G, and S180T/Q289G variants were 4.37 ± 0.28 nm, 4.32 ± 0.34 nm, and 4.88 ± 0.41 nm, respectively (mean ± SEM, n = 15 each) (Figure 10). These values fall within the expected range of 4 nm to 6 nm and are in excellent agreement with the crystallographic dimensions of the monomeric protein (Figure 9), supporting their monomeric state in solution. Minor peaks corresponding to larger particles were observed in the intensity distribution for some samples; however, their contribution to the overall number distribution was negligible (less than 1%), indicating trace aggregation or scattering artifacts.

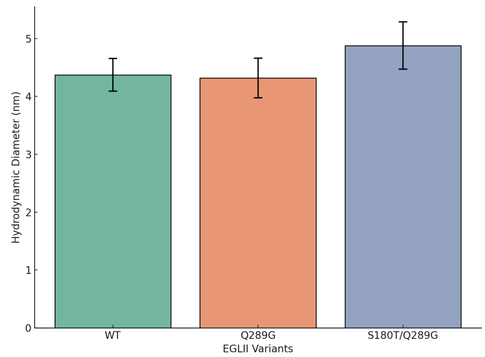

Figure 10: Estimated hydrodynamic diameters of EGLII variants under native buffer conditions (Range 1)

6.3.2. *DLS after thermal stress*

DLS measurements were performed after one hour of incubation at 75 to assess the thermal stability of the EGLII variants. The average diameters (Range 1) of the WT, Q289G, and S180T/Q289G variants increased to 7.70 ± 1.40 nm, 5.31 ± 0.70 nm, and 6.50 ± 0.72 nm, respectively (mean ± SEM, n = 8–10) (Figure 11). The Q289G and S180T/Q289G variants remained within the expected monomeric size range, while the WT variant showed a marked increase in hydrodynamic diameter, suggesting thermal-induced expansion or partial aggregation. Analysis of particle count distributions showed no detectable particles in the aggregate range (Range 2) for Q289G, and only a single S180T/Q289G replicate showed a trace amount of larger species, which was negligible by number. In contrast, 2 out of 8 WT samples contained measurable populations in the aggregate size range. Furthermore, a comparison of % mass in the monomeric size range (Range 1) revealed a significant drop in the WT variant (from ~99% in buffer to 81.7 % after heating), whereas the Q289G and S180T/Q289G variants maintained higher monomeric mass percentages of 93.9 % and 94.7 %, respectively. These results indicate enhanced thermal stability of the two variants.

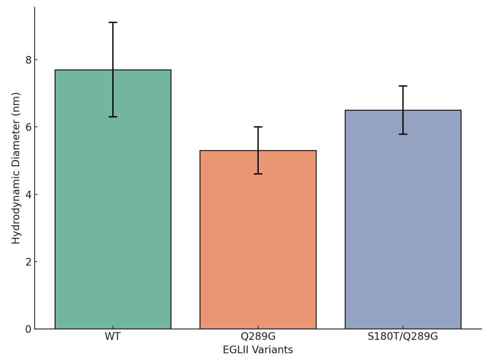

Figure 11: Estimated hydrodynamic diameters of EGLII variants after one hour incubation at 75 °C (Range 1)

6.3.3. *DLS under high salt conditions*

To evaluate salt stability, DLS measurements were conducted for EGLII variants at two salt concentration (1 M and 3 M NaCl). At both salt concentrations, all three variants maintained monomeric sizes, and no particles were detected in the aggregate size range (Range 2) in any sample.In 1 M NaCl, the average hydrodynamic diameters for WT, Q289G, and S180T/Q289G were 4.38 ± 0.61 nm, 5.75 ± 0.39 nm, and 6.40 ± 0.17 nm, respectively

(mean ± SEM, $n = 8$). In 3 M NaCl, the diameters were 5.07 ± 0.44 nm, 5.97 ± 0.63 nm, and 6.07 ± 0.82 nm, respectively ($n = 8$–9) (Figure 12. All values fall within or slightly above the expected monomeric range based on the crystallographic dimensions (maximum extent ~5.5–6 nm). Mass distribution in the range (Range 1) remained high for all variants at 3 M NaCl: 97.2 % (WT), 98.9 % (Q289G), and 98.3 % (S180T/Q289G). These results confirm that all EGLII variants remain monomeric even under extreme ionic strength. Both Q289G and S180T/Q289G demonstrate excellent salt stability, with Q289G showing the most consistent behavior across conditions.

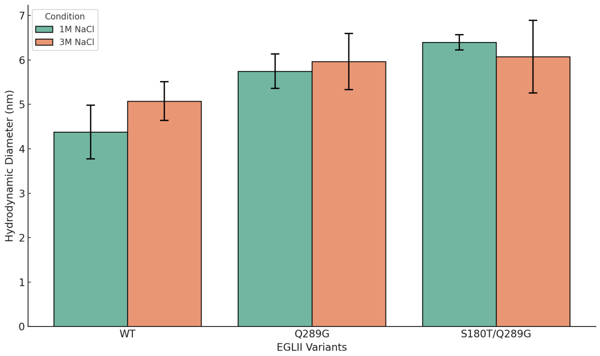

Figure 12: Estimated hydrodynamic diameters of EGLII variants in presence of high salt concentrations (Range 1)

7. SDS-PAGE analysis of predicted EGLII variants from FoldX and EVmutation (single and double substituted)

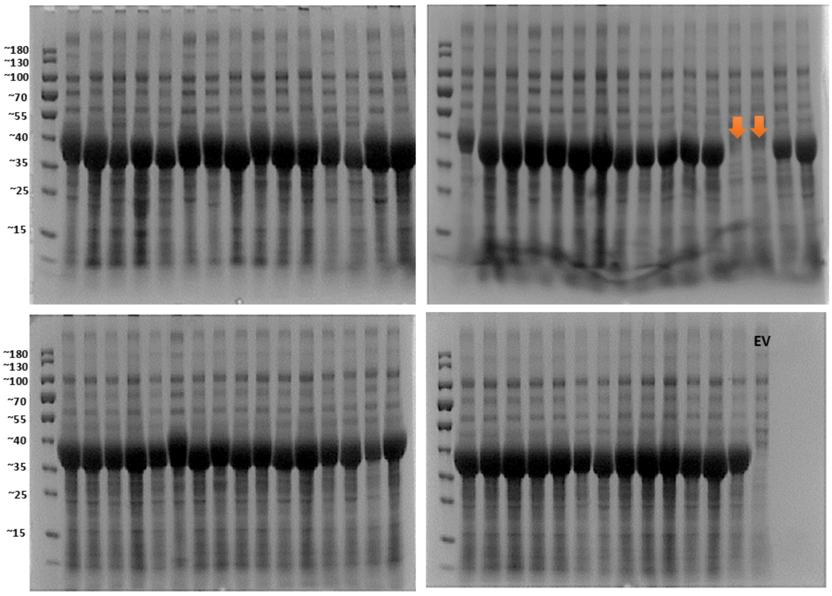

Figure 13: SDS-PAGE analysis of predicted variants from FoldX and EVmutation (single- and double-substituted)

"EV" represents the empty vector. Our protein of interest, approximately 35 kDa, is represented by a prominent band on the gel. Notably, the two variants indicated by arrows (T110N and N299S) exhibited no expression; consequently, these variants were re-transformed, and all analyses were repeated for them.

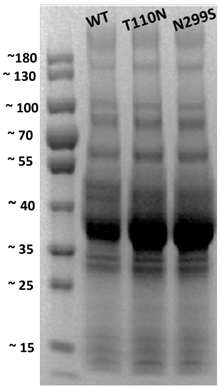

Figure 14: SDS-PAGE analysis of EGLII wild-type and variants T110N and N299S.

8. Half-lives of predicted EGLII variants from FoldX and EVmutation (single and double substituted)

Table 7: Half-lives of the predicted EGLII variants by FoldX and EVmutation.

The variants depicted as N/A exhibited very little initial activity (without incubation at 75 °C), making half-life determination not applicable.

**FoldX Single-substituted variants**		**Evmutation Single-substituted variants**		**Evmutation Double-substituted variants**	
**Variants**	**Half-Lives (minutes)**	**Variants**	**Half-Lives (minutes)**	**Variants**	**Half-Lives (minutes)**
S280L	54 ± 8	Q289G	104 ± 7	P111I/Q289G	122 ± 8
S280M	54 ± 2	S180T	63 ± 4	S180T/Q289G	121 ± 15
**WT**	**40 ± 2**	V226I	60 ± 7	P111V/Q289G	118 ± 7
S114D	36 ± 2	P111I	54 ± 6	E81A/Q289G	86 ± 16
E205Q	17 ± 1	T236A	51 ± 5	D164A/Q289G	84 ± 14
E20M	15 ± 1	P111V	49 ± 2	T110N/Q289G	83 ± 5
E20L	15 ± 1	Q239K	49 ± 3	V226I/Q289G	76 ± 9
D138L	14 ± 1	D164A	48 ± 6	T236Q/Q289G	60 ± 3
E20I	14 ± 1	T236Q	48 ± 3	Q289G/N299S	59 ± 3
D138A	N/A	A153D	45 ± 5	D164G/Q289G	54 ± 3
D138C	N/A	E81A	44 ± 4	P111I/V226I	54 ± 6
D138N	N/A	L201K	43 ± 3	P111I/D164A	50 ± 2
D138S	N/A	D164G	43 ± 3	P111V/V226I	48 ± 2
D138T	N/A	Q289A	43 ± 7	D164A/V226I	46 ± 3
D138V	N/A	V150L	42 ± 4	A178S/Q289G	46 ± 4
E175M	N/A	**WT**	**40 ± 2**	**WT**	**40 ± 2**
E205A	N/A	Y188V	40 ± 2	P111I/Q289A	40 ± 5
E205C	N/A	S123T	34 ± 5	V226I/Q289A	39 ± 4
E205L	N/A	T110N	29 ± 2	P111I/A178S	37 ± 2
E205M	N/A	A178S	26 ± 4	A178S/V226I	24 ± 2
G181R	N/A	N299S	20 ± 2	D164A/A178S	22 ± 2

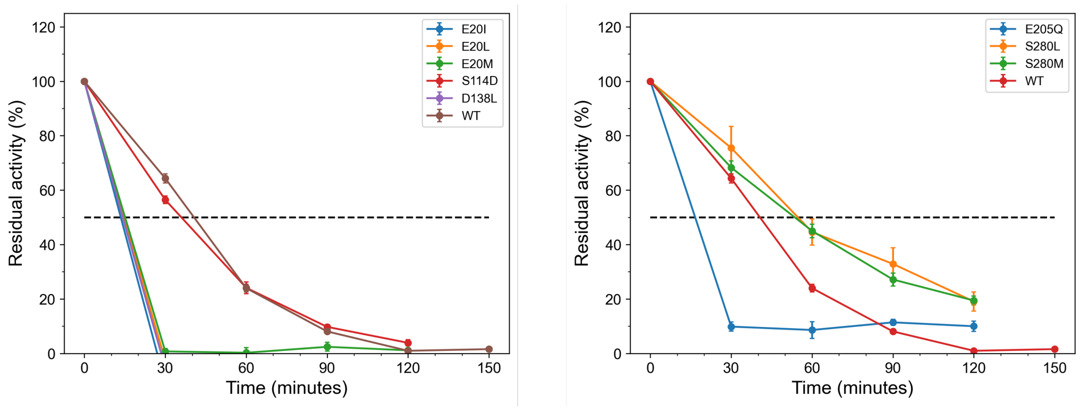

Figure 15: Half-lives of single-substituted EGLII variants predicted by FoldX.

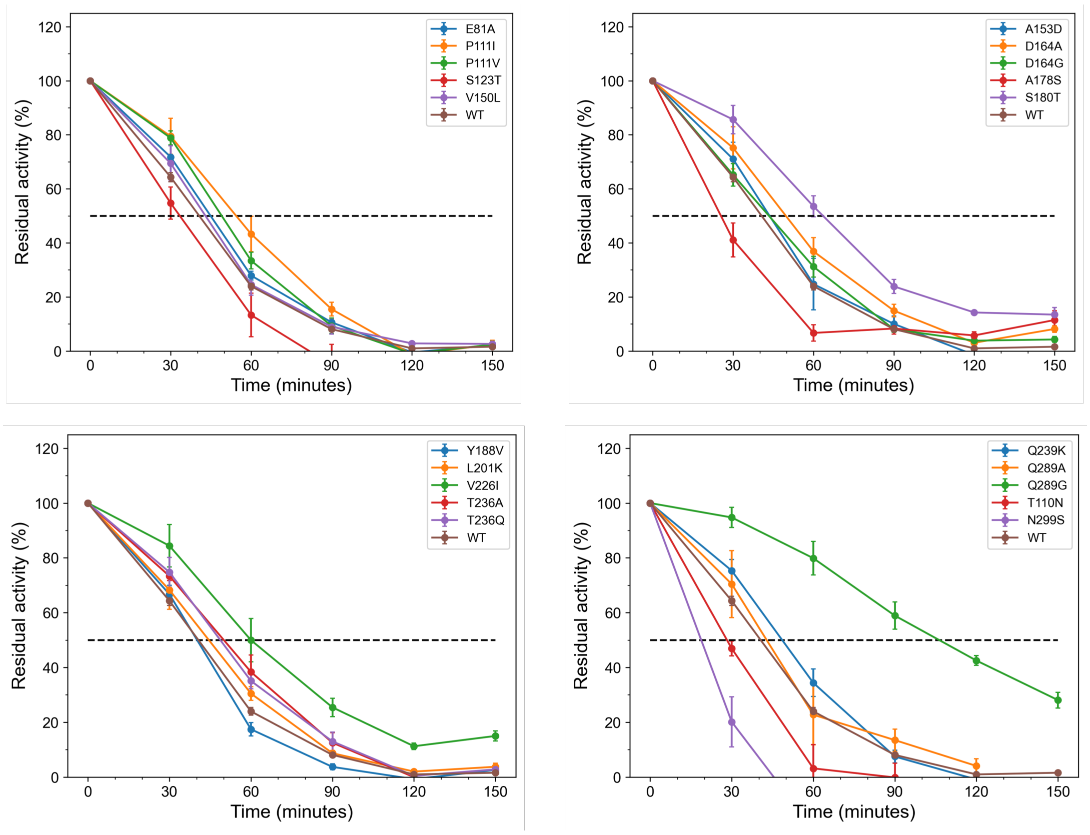

Figure 16: Half-lives of single-substituted EGLII variants predicted by EVmutation

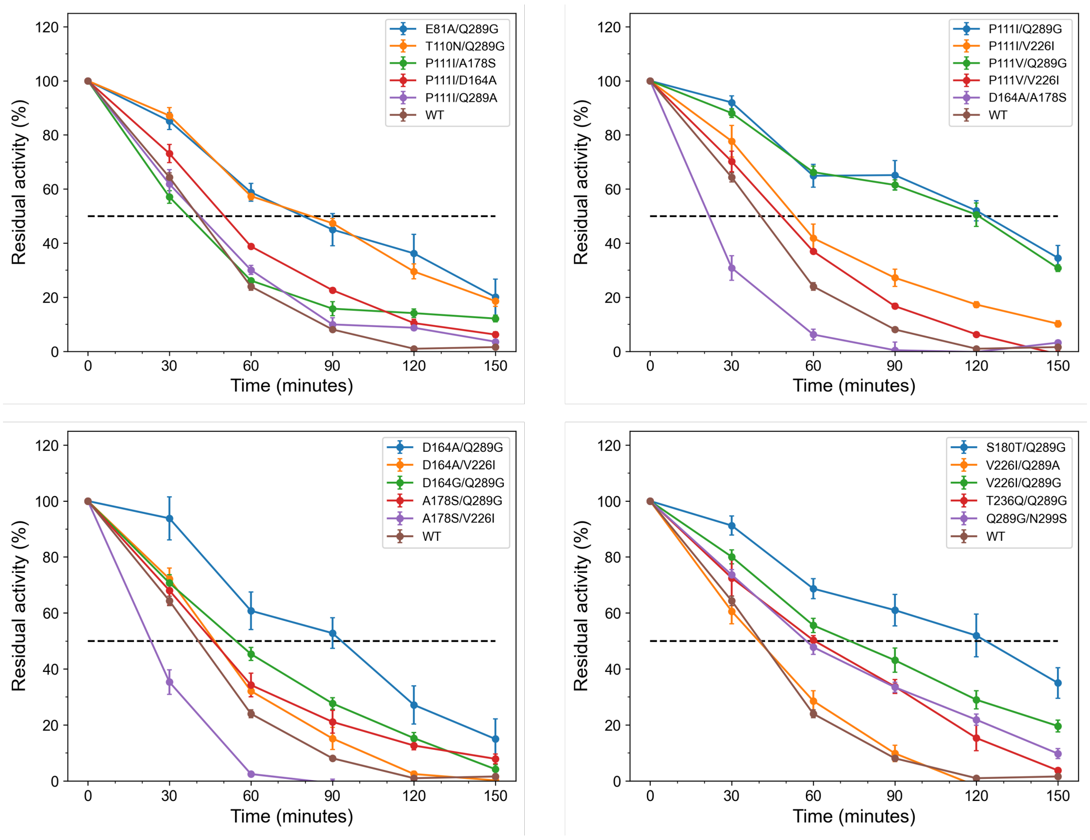

Figure 17: Half-lives of double-substituted EGLII variants predicted by EVmutation

9. Enzymatic activity of predicted EGLII variants without thermal stress

All predicted EGLII variants by FoldX and EVmutation were assessed by a hydrolytic assay using solubilized Azo-CM-Cellulose, as detailed in the Materials and Methods section.

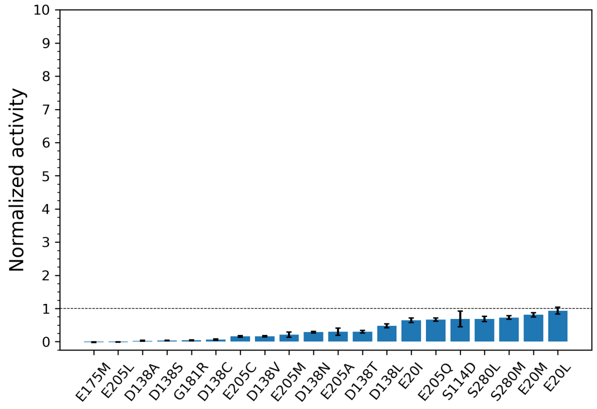

Figure 18: Normalized enzymatic activity of FoldX-predicted single-substituted variants without thermal stress.

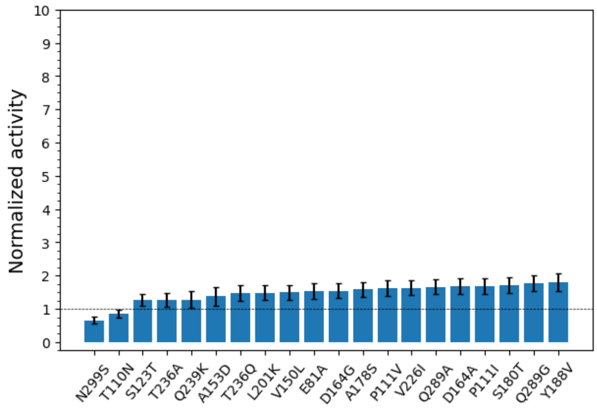

Figure 19: Normalized enzymatic activity of EVmutation-predicted single-substituted variants without thermal stress.

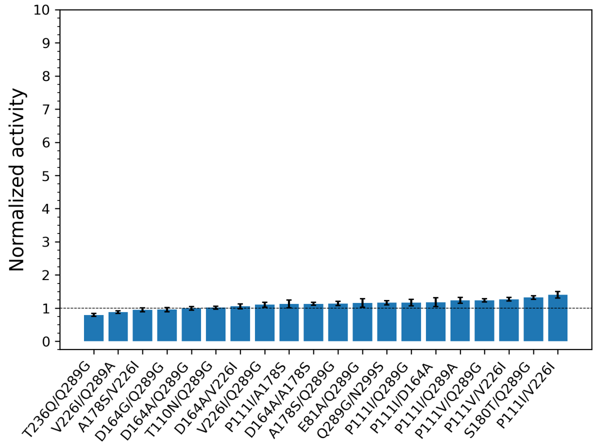

Figure 20: Normalized enzymatic activity of EVmutation-predicted double-substituted variants without thermal stress.

10. Enzymatic activity of predicted EGLII variants after thermal stress at 76 °C

In addition to incubation at 75 °C, all predicted EGLII variants were also subjected to thermal stress by incubation at 76 °C. Although the results at 76 °C were similar to those at 75 °C, the observed fold improvement was greater at elevated temperature.

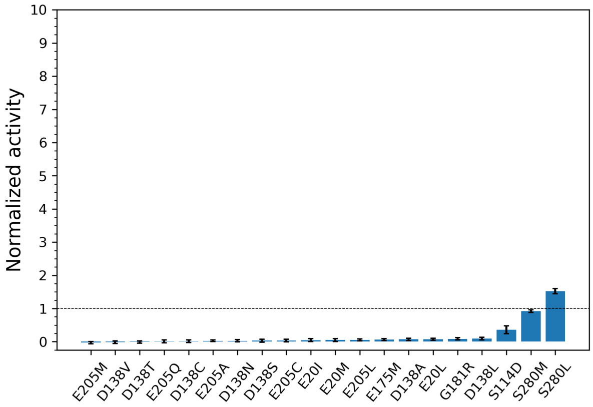

Figure 21: Normalized enzymatic activity of FoldX-predicted single-substituted variants after 1-hour incubation at 76 °C.

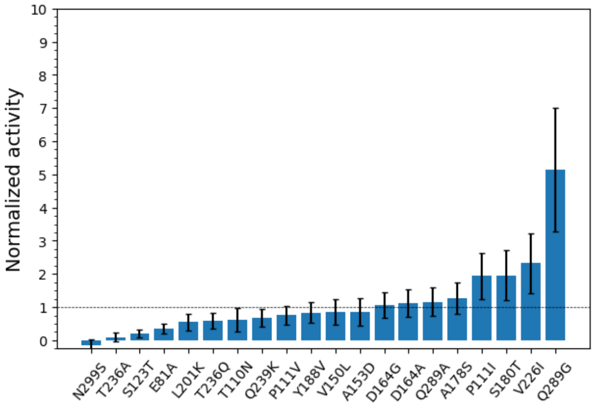

Figure 22: Normalized enzymatic activity of EVmutation-predicted single-substituted variants after 1-hour incubation at 76 °C.

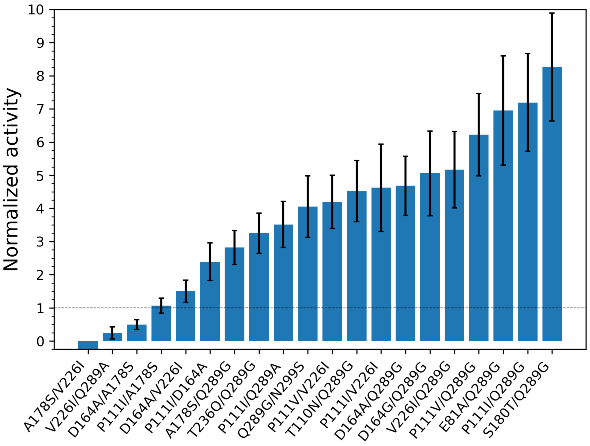

Figure 23: Normalized enzymatic activity of EVmutation-predicted double-substituted variants after 1-hour incubation at 76 °C.

11. Thermal unfolding curves of predicted EGLII variants from FoldX and EVmutation (single and double substituted)

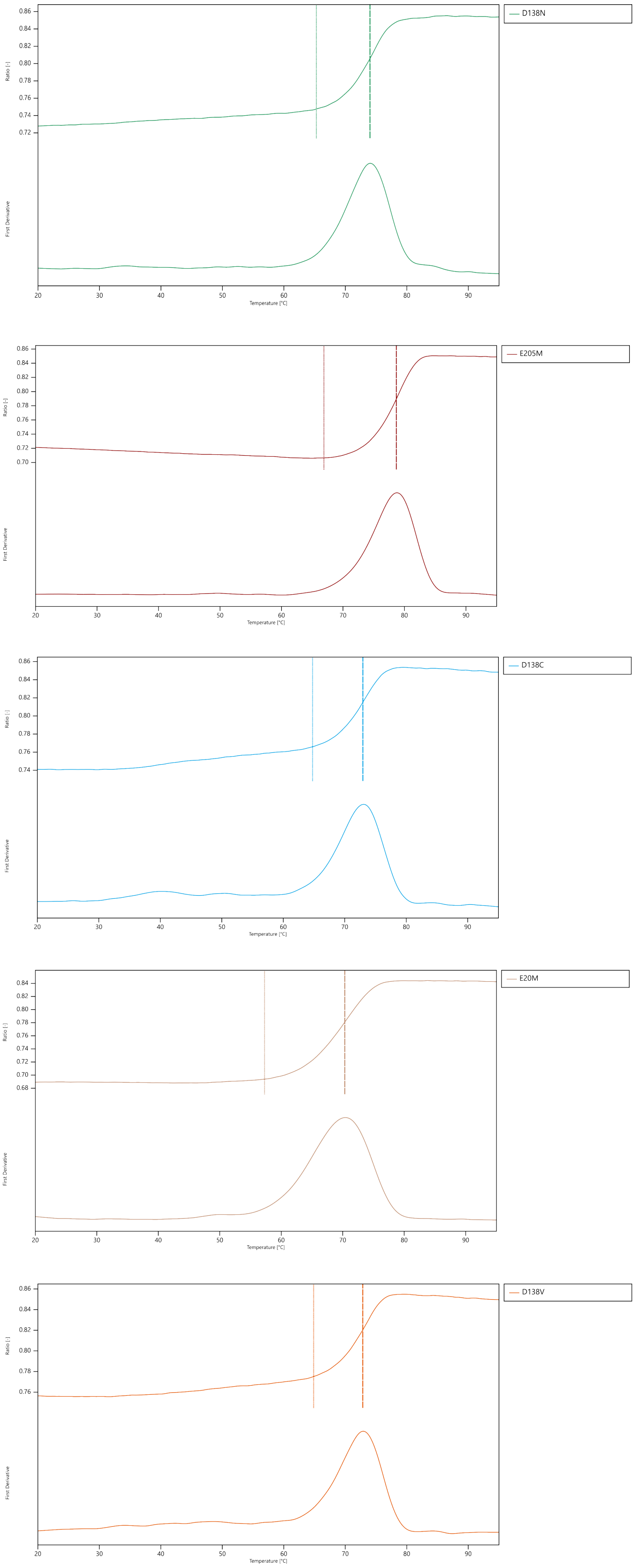

Figure 24: Thermal unfolding curves of FoldX single-substituted variants

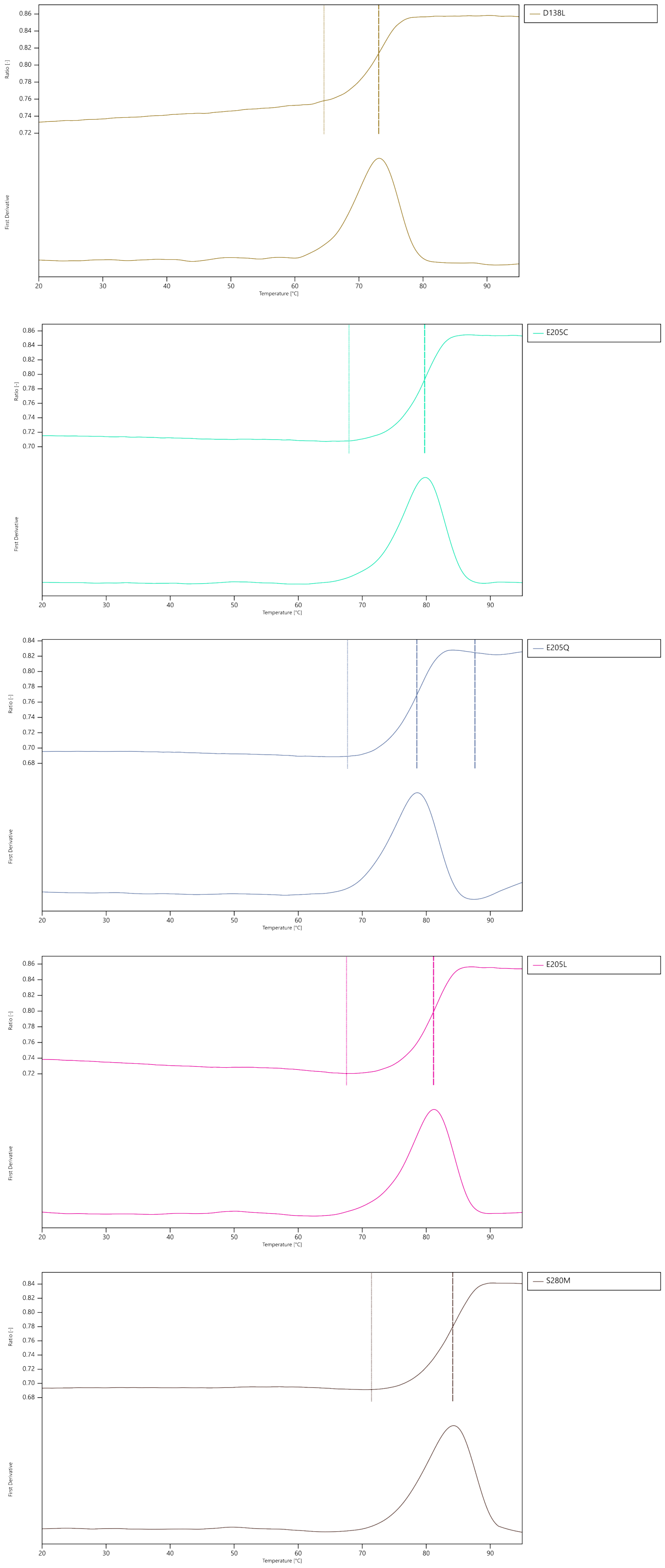

Figure 25: Thermal unfolding curves of FoldX single-substituted variants

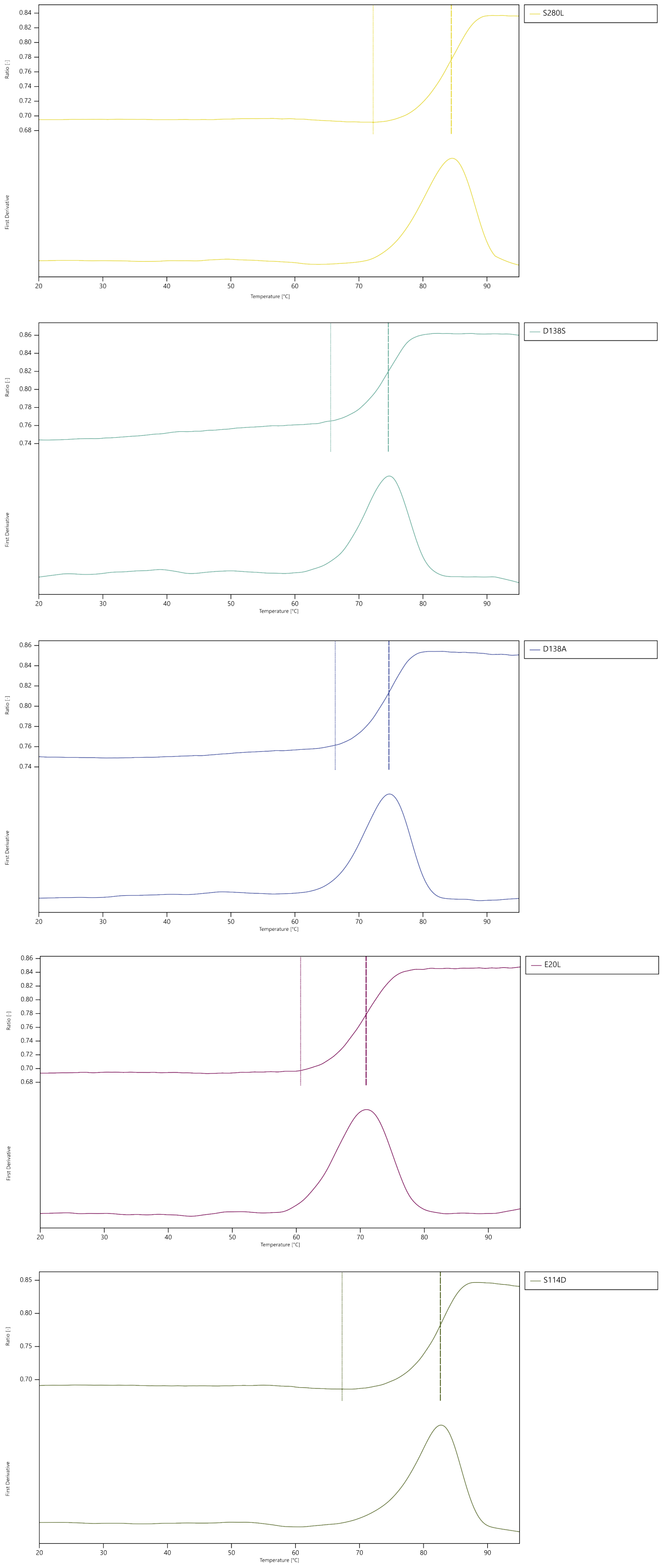

Figure 26: Thermal unfolding curves of FoldX single-substituted variants

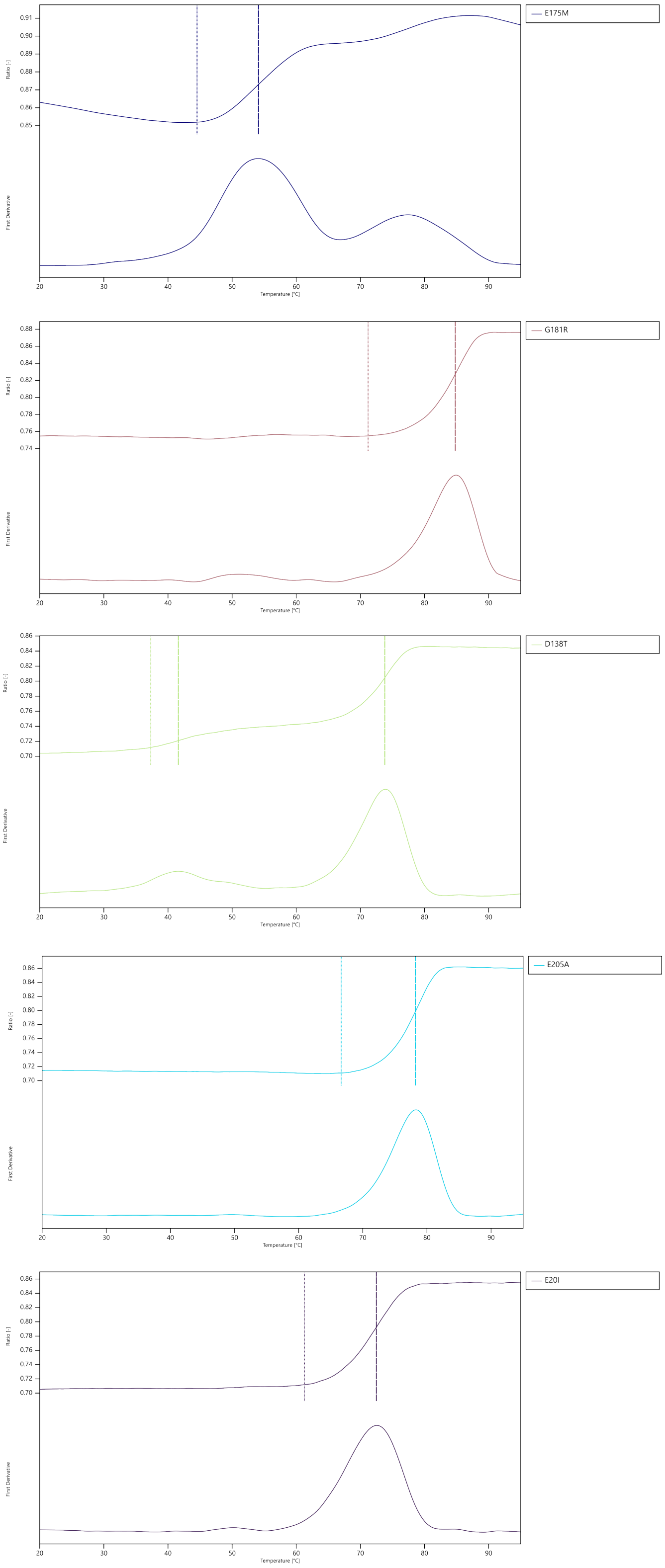

Figure 27: Thermal unfolding curves of FoldX single-substituted variants

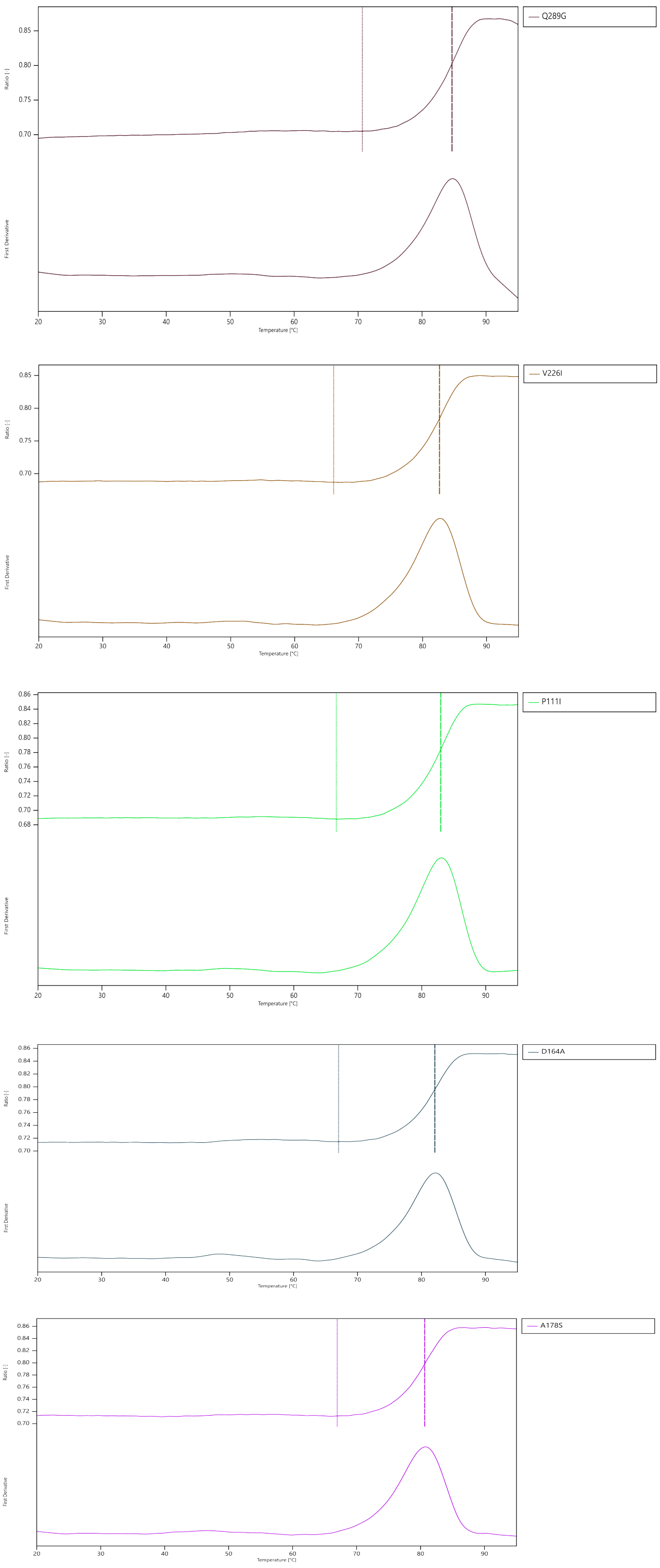

Figure 28: Thermal unfolding curves of EVmutation single-substituted variants

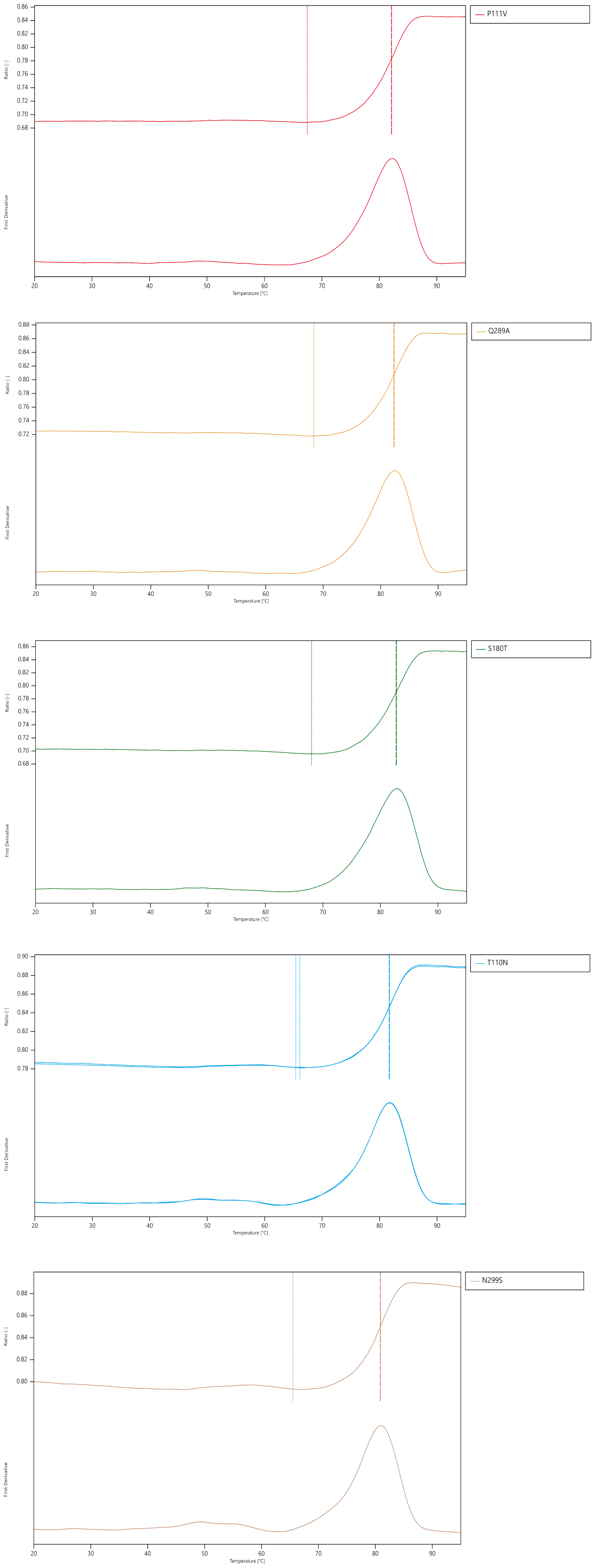

Figure 29: Thermal unfolding curves of EVmutation single-substituted variants

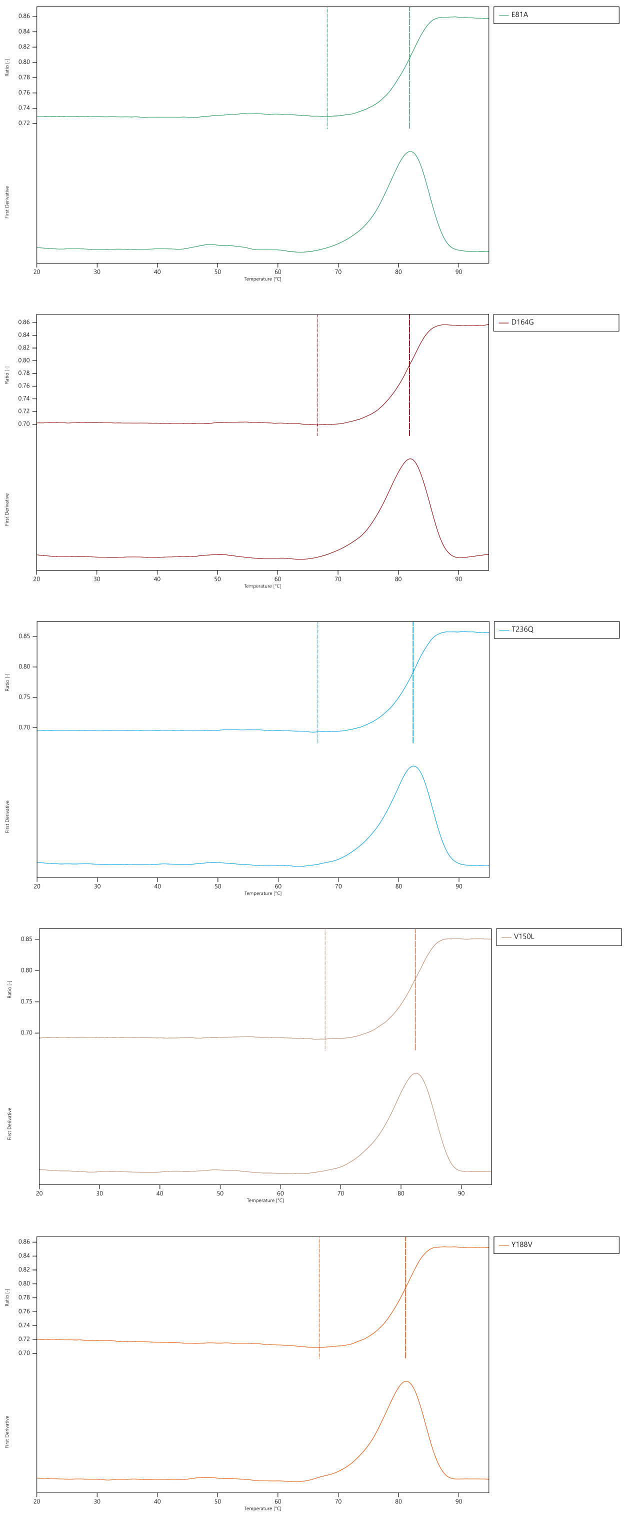

Figure 30: Thermal unfolding curves of EVmutation single-substituted variants

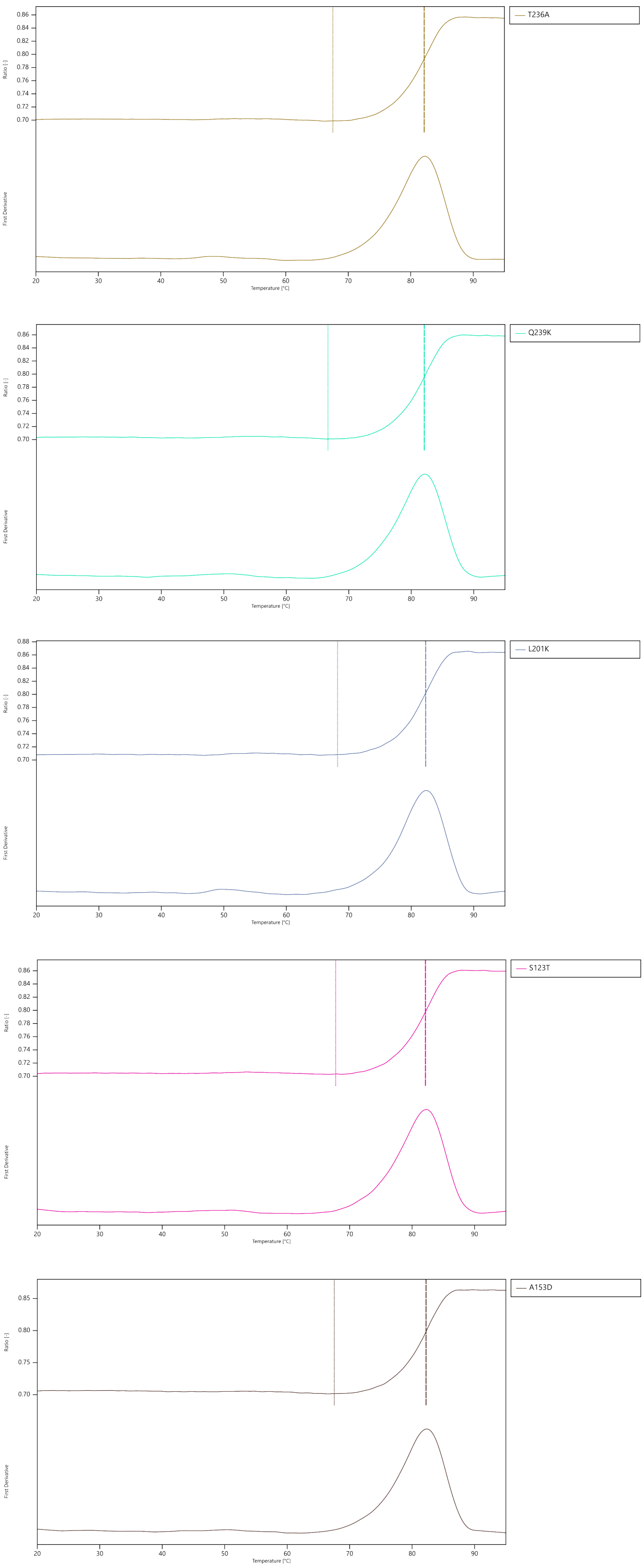

Figure 31: Thermal unfolding curves of EVmutation single-substituted variants

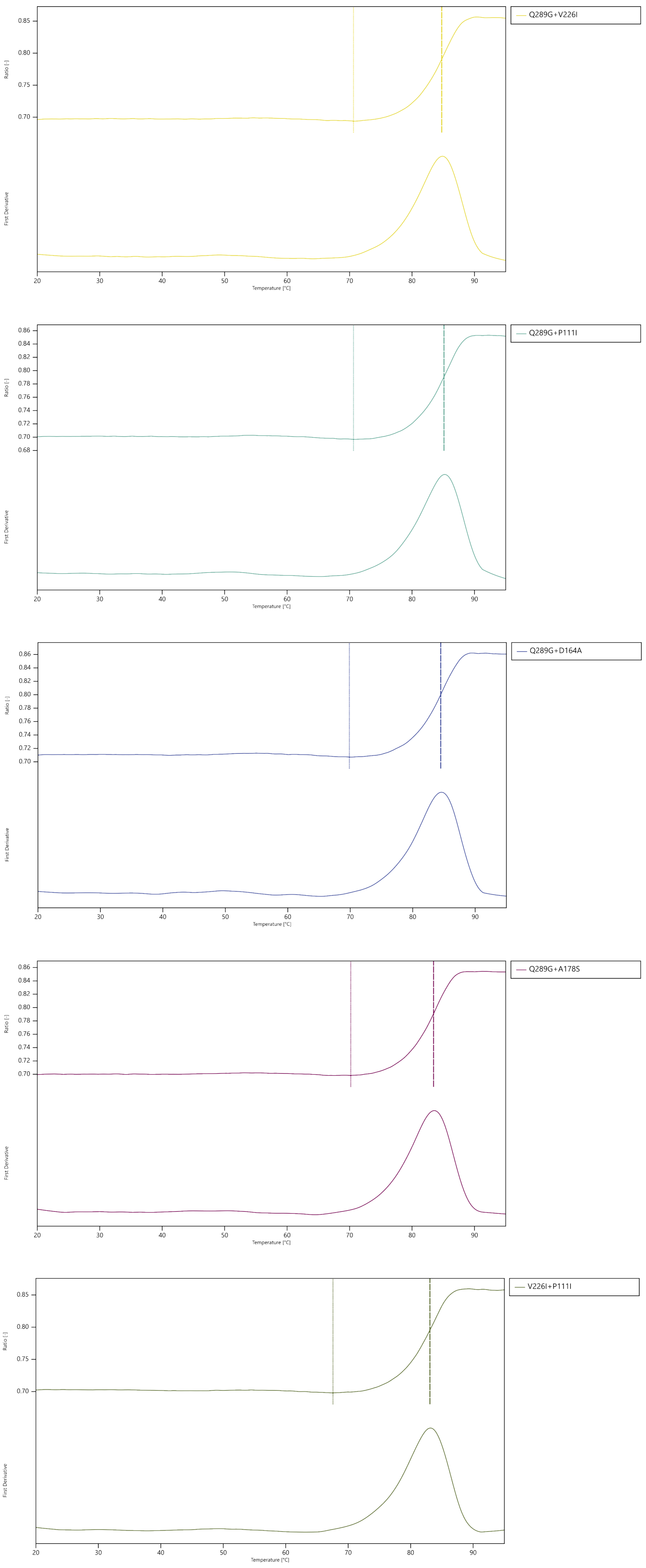

Figure 32: Thermal unfolding curves of EVmutation double-substituted variants

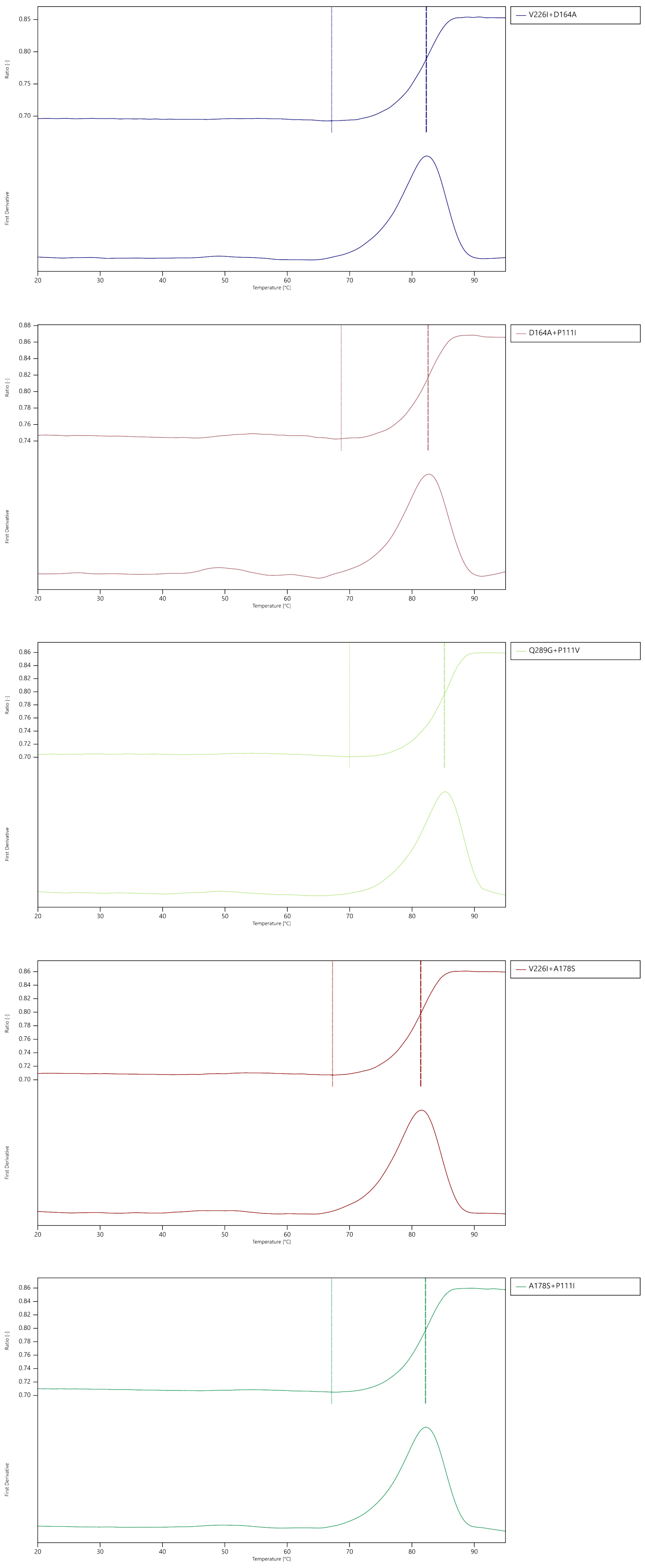

Figure 33: Thermal unfolding curves of EVmutation double-substituted variants

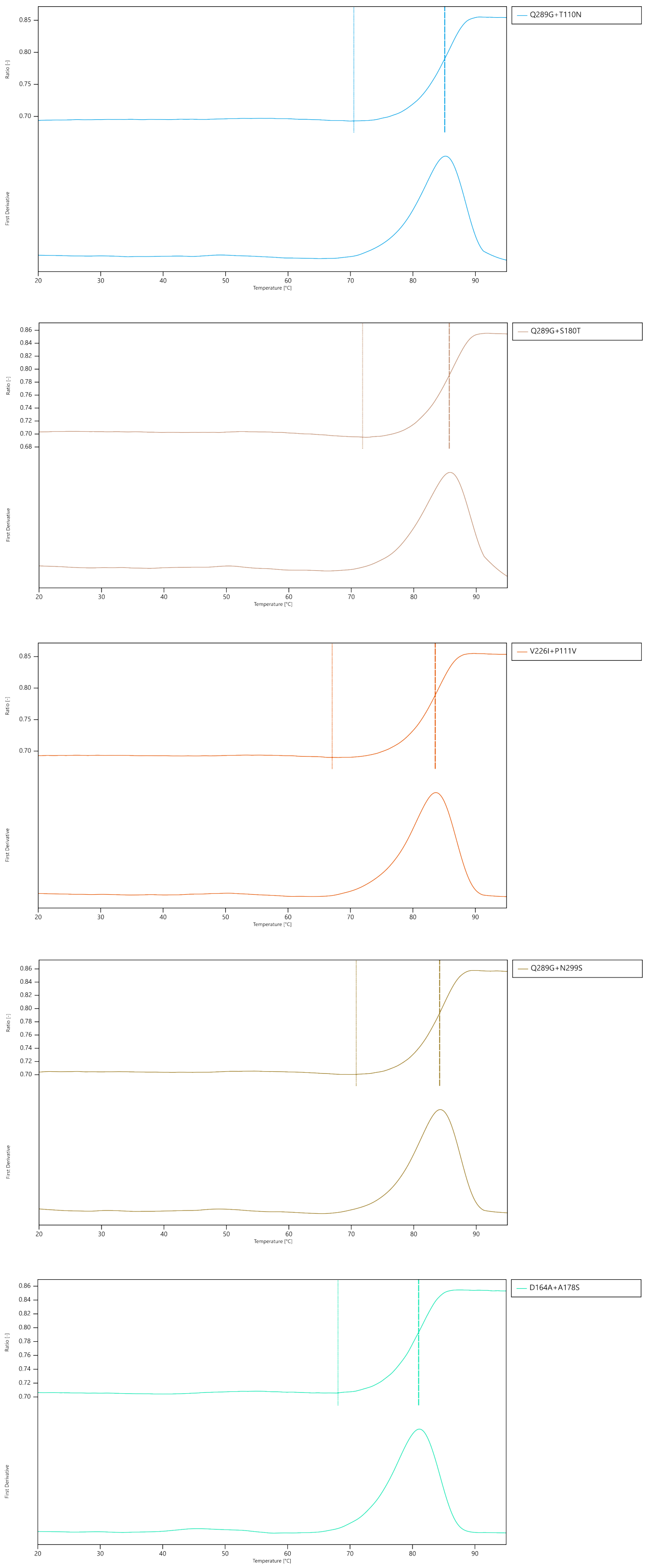

Figure 34: Thermal unfolding curves of EVmutation double-substituted variants

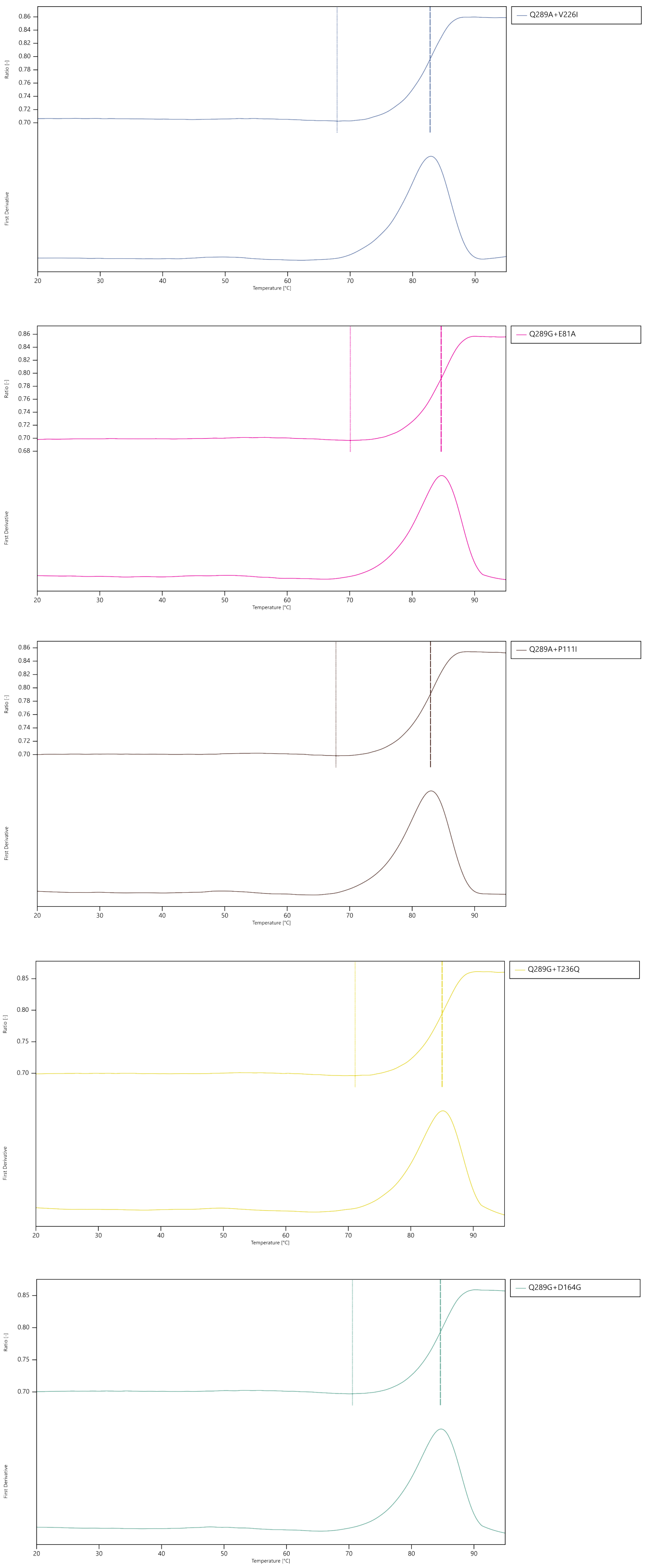

Figure 35: Thermal unfolding curves of EVmutation double-substituted variants

12. ConSurf analysis of predicted EGLII positions (FoldX and EVmutation)

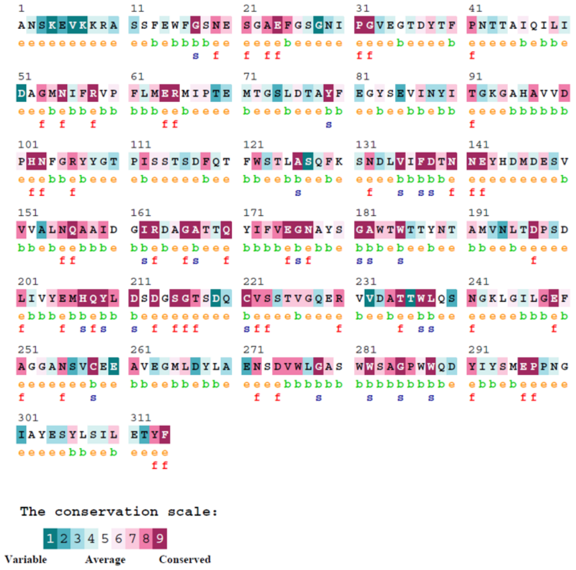

Figure 36: ConSurf analysis of EGLII

Table 8: Conservation and exposure of predicted EGLII positions

Position	Method	Residue	Conservation Score	Conservation Level	Exposure
20	FoldX	E	7	Moderate	Exposed
114	FoldX	S	6	Moderate	Exposed
138	FoldX	D	9	High	Buried
175	FoldX	E	9	High	Exposed
181	FoldX	G	9	High	Buried
205	FoldX	E	8	High	Exposed
280	FoldX	S	5	Moderate	Buried
81	EVmutation	E	3	Low	Exposed
110	EVmutation	T	3	Low	Exposed
111	EVmutation	P	4	Low	Exposed
123	EVmutation	S	3	Low	Exposed
150	EVmutation	V	5	Moderate	Buried
153	EVmutation	A	2	Low	Exposed
164	EVmutation	D	4	Low	Buried
178	EVmutation	A	6	Moderate	Buried
180	EVmutation	S	6	Moderate	Exposed
188	EVmutation	Y	5	Moderate	Buried
201	EVmutation	L	8	High	Exposed
226	EVmutation	V	7	Moderate	Buried
236	EVmutation	T	1	Low	Exposed
239	EVmutation	Q	5	Moderate	Exposed
289	EVmutation	Q	4	Low	Buried
299	EVmutation	N	4	Low	Exposed

13. References

[1] H. Nakazawa, K. Okada, T. Onodera, W. Ogasawara, H. Okada, Y. Morikawa, Directed evolution of endoglucanase iii (cel12a) from trichoderma reesei, Applied microbiology and biotechnology 83 (2009) 649–657.

[2] M. Anbar, R. Lamed, E. A. Bayer, Thermostability enhancement of clostridium thermocellum cellulosomal endoglucanase cel8a by a single glycine substitution, ChemCatChem 2 (2010) 997–1003.

[3] X.-J. Wang, Y.-J. Peng, L.-Q. Zhang, A.-N. Li, D.-C. Li, Directed evolution and structural prediction of cellobiohydrolase ii from the thermophilic fungus chaetomium thermophilum, Applied microbiology and biotechnology 95 (2012) 1469–1478.

[4] I. Wu, F. H. Arnold, Engineered thermostable fungal cel6a and cel7a cellobiohydrolases hydrolyze cellulose efficiently at elevated temperatures, Biotechnology and Bioengineering 110 (2013) 1874–1883.

[5] L. Cao, S. Li, X. Huang, Z. Qin, W. Kong, W. Xie, Y. Liu, Enhancing the thermostability of highly active and glucose-tolerant β-glucosidase ks5a7 by directed evolution for good performance of three properties, Journal of agricultural and food chemistry 66 (2018) 13228–13235.

[6] S. Yoav, J. Stern, O. Salama-Alber, F. Frolow, M. Anbar, A. Karpol, Y. Hadar, E. Morag, E. A. Bayer, Directed evolution of clostridium thermocellum β-glucosidase a towards enhanced thermostability, International Journal of Molecular Sciences 20 (2019) 4701.

[7] S. P. Voutilainen, H. Boer, M. Alapuranen, J. Jänis, J. Vehmaanperä, A. Koivula, Improving the thermostability and activity of melanocarpus albomyces cellobiohydrolase cel7b, Applied microbiology and biotechnology 83 (2009) 261–272.

[8] J. Zhang, H. Shi, L. Xu, X. Zhu, X. Li, Site-directed mutagenesis of a hyperthermophilic endoglucanase cel12b from thermotoga maritima based on rational design, PLoS One 10 (2015) e0133824.

[9] X. Chen, W. Li, P. Ji, Y. Zhao, C. Hua, C. Han, Engineering the conserved and noncatalytic residues of a thermostable β-1, 4-endoglucanase to improve specific activity and thermostability, Scientific reports 8 (2018) 2954.

[10] C. Han, Q. Wang, Y. Sun, R. Yang, M. Liu, S. Wang, Y. Liu, L. Zhou, D. Li, Improvement of the catalytic activity and thermostability of a hyperthermostable endoglucanase by optimizing n-glycosylation sites, Biotechnology for biofuels 13 (2020) 1–11.

[11] C. Han, Y. Liu, M. Liu, S. Wang, Q. Wang, Improving the thermostability of a thermostable endoglucanase from chaetomium thermophilum by engineering the conserved noncatalytic residue and n-glycosylation site, International Journal of Biological Macromolecules 164 (2020) 3361–3368.

[12] A. Bashirova, S. Pramanik, P. Volkov, A. Rozhkova, V. Nemashkalov, I. Zorov, A. Gusakov, A. Sinitsyn, U. Schwaneberg, M. D. Davari, Disulfide bond engineering of an endoglucanase from penicillium verruculosum to improve its thermostability, International Journal of Molecular Sciences 20 (2019) 1602.

[13] A. S. Dotsenko, S. Pramanik, A. V. Gusakov, A. M. Rozhkova, I. N. Zorov, A. P. Sinitsyn, M. D. Davari, U. Schwaneberg, Critical effect of proline on thermostability of endoglucanase ii from penicillium verruculosum, Biochemical engineering journal 152 (2019) 107395.

[14] A. S. Dotsenko, G. S. Dotsenko, A. M. Rozhkova, I. N. Zorov, A. P. Sinitsyn, Rational design and structure insights for thermostability improvement of penicillium verruculosum cel7a cellobiohydrolase, Biochimie 176 (2020) 103–109.

[15] C. M. Dana, P. Saija, S. M. Kal, M. B. Bryan, H. W. Blanch, D. S. Clark, Biased clique shuffling reveals stabilizing mutations in cellulase cel7a, Biotechnology and bioengineering 109 (2012) 2710–2719.

[16] Y. Ito, A. Ikeuchi, C. Imamura, Advanced evolutionary molecular engineering to produce thermostable cellulase by using a small but efficient library, Protein Engineering, Design & Selection 26 (2013) 73–79.

[17] A. Mitrovic, K. Flicker, G. Steinkellner, K. Gruber, C. Reisinger, G. Schirrmacher, A. Camattari, A. Glieder, Thermostability improvement of endoglucanase cel7b from hypocrea pseudokoningii, Journal of Molecular Catalysis B: Enzymatic 103 (2014) 16–23.

[18] C.-J. Chang, C.-C. Lee, Y.-T. Chan, D. L. Trudeau, M.-H. Wu, C.-H. Tsai, S.-M. Yu, T.-H. D. Ho, A. H.-J. Wang, C.-D. Hsiao, et al., Exploring the mechanism responsible for cellulase thermostability by structure-guided recombination, PLoS One 11 (2016) e0147485.

[19] F. Goedegebuur, L. Dankmeyer, P. Gualfetti, S. Karkehabadi, H. Hansson, S. Jana, V. Huynh, B. R. Kelemen, P. Kruithof, E. A. Larenas, et al., Improving the thermal stability of cellobiohydrolase cel7a from hypocrea jecorina by directed evolution, Journal of Biological Chemistry 292 (2017) 17418–17430.

[20] F. Zheng, J. V. Vermaas, J. Zheng, Y. Wang, T. Tu, X. Wang, X. Xie, B. Yao, G. T. Beckham, H. Luo, Activity and thermostability of gh5 endoglucanase chimeras from mesophilic and thermophilic parents, Applied and environmental microbiology 85 (2019) e02079–18.

[21] F. Contreras, M. J. Thiele, S. Pramanik, A. M. Rozhkova, A. S. Dotsenko, I. N. Zorov, A. P. Sinitsyn, M. D. Davari, U. Schwaneberg, Knowvolution of a gh5 cellulase from penicillium verruculosum to improve thermal stability for biomass degradation, ACS Sustainable Chemistry & Engineering 8 (2020) 12388–12399.

[22] F. Contreras, C. Nutschel, L. Beust, M. D. Davari, H. Gohlke, U. Schwaneberg, Can constraint network analysis guide the identification phase of knowvolution? a case study on improved thermostability of an endo-β-glucanase, Computational and structural biotechnology journal 19 (2021) 743–751.

[23] W. Wang, B. A. Malcolm, Two-stage pcr protocol allowing introduction of multiple mutations, deletions and insertions using quikchangetm site-directed mutagenesis, Biotechniques 26 (1999) 680–682.

[24] S. R. Eddy, Accelerated profile hmm searches, PLoS computational biology 7 (2011) e1002195.

[25] Uniprot: the universal protein knowledgebase in 2023, Nucleic acids research 51 (2023) D523–D531.

[26] A. Bateman, M.-J. Martin, S. Orchard, M. Magrane, S. Ahmad, E. Alpi, E. H. Bowler-Barnett, R. Britto, H. Bye-A-Jee, A. Cukura, P. Denny, T. Dogan, T. Ebenezer, J. Fan, P. Garmiri, L. J. da Costa Gonzales, E. Hatton-Ellis, A. Hussein, A. Ignatchenko, G. Insana, R. Ishtiaq, V. Joshi, D. Jyothi, S. Kandasaamy, A. Lock, A. Luciani, M. Lugaric, J. Luo, Y. Lussi, A. MacDougall, F. Madeira, M. Mahmoudy, A. Mishra, K. Moulang, A. Nightingale, S. Pundir, G. Qi, S. Raj, P. Raposo, D. L. Rice, R. Saidi, R. Santos, E. Speretta, J. Stephenson, P. Totoo, E. Turner, N. Tyagi, P. Vasudev, K. Warner, X. Watkins, R. Zaru, H. Zellner, A. J. Bridge, L. Aimo, G. Argoud-Puy, A. H. Auchincloss, K. B. Axelsen, P. Bansal, D. Baratin, T. M. Batista Neto, M.-C. Blatter, J. T. Bolleman, E. Boutet, L. Breuza, B. C. Gil, C. Casals-Casas, K. C. Echioukh, E. Coudert, B. Cuche, E. de Castro, A. Estreicher, M. L. Famiglietti, M. Feuermann, E. Gasteiger, P. Gaudet, S. Gehant, V. Gerritsen, A. Gos, N. Gruaz, C. Hulo, N. Hyka-Nouspikel, F. Jungo, A. Kerhornou, P. Le Mercier, D. Lieberherr, P. Masson, A. Morgat, V. Muthukrishnan, S. Paesano, I. Pedruzzi, S. Pilbout, L. Pourcel, S. Poux, M. Pozzato, M. Pruess, N. Redaschi, C. Rivoire, C. J. A. Sigrist, K. Sonesson, S. Sundaram, C. H. Wu, C. N. Arighi, L. Arminski, C. Chen, Y. Chen, H. Huang, K. Laiho, P. McGarvey, D. A. Natale, K. Ross, C. R. Vinayaka, Q. Wang, Y. Wang, J. Zhang, Uniprot: the universal protein knowledgebase in 2023, Nucleic Acids Research 51 (2022) D523–D531. URL: `http://dx.doi.org/10.1093/nar/gkac1052`. doi:`10.1093/nar/gkac1052`.

[27] T. A. Hopf, J. B. Ingraham, F. J. Poelwijk, C. P. Schärfe, M. Springer, C. Sander, D. S. Marks, Mutation effects predicted from sequence co-variation, Nature biotechnology 35 (2017) 128–135.

[28] V. J. Frauenkron-Machedjou, A. Fulton, L. Zhu, C. Anker, M. Bocola, K.-E. Jaeger, U. Schwaneberg, Towards understanding directed evolution: more than half of all amino acid positions contribute to ionic liquid resistance of bacillus subtilis lipase a, ChemBioChem 16 (2015) 937–945.
